# Role of *BicDR* in bristle shaft construction and support of *BicD* functions

**DOI:** 10.1242/jcs.261408

**Published:** 2024-01-31

**Authors:** Aleksandra Jejina, Yeniffer Ayala, Dirk Beuchle, Thomas Höhener, Ruth E. Dörig, Paula Vazquez-Pianzola, Greco Hernández, Beat Suter

**Affiliations:** ^1^ Institute of Cell Biology, University of Bern, CH-3012 Bern, Switzerland; ^2^ Graduate School for Cellular and Biomedical Sciences, University of Bern, CH-3012 Bern, Switzerland; ^3^ Laboratory of Translation and Cancer, Unit of Biomedical Research on Cancer, Instituto Nacional de Cancerologıá (INCan), 14080 Tlalpan, Mexico City, Mexico

**Keywords:** *Drosophila*, Bicaudal-D, BicD, BicD-related, Bristle formation, Rab6, Spn-F, Microtubule vesicle transport

## Abstract

Cell polarization requires asymmetric localization of numerous mRNAs, proteins and organelles. The movement of cargo towards the minus end of microtubules mostly depends on cytoplasmic dynein motors. In the dynein–dynactin–Bicaudal-D transport machinery, Bicaudal-D (BicD) links the cargo to the motor. Here, we focus on the role of *Drosophila BicD-related* (*BicDR, CG32137*) in the development of the long bristles. Together with *BicD*, it contributes to the organization and stability of the actin cytoskeleton in the not-yet-chitinized bristle shaft. *BicD* and *BicDR* also support the stable expression and distribution of Rab6 and Spn-F in the bristle shaft, including the distal tip localization of Spn-F, pointing to the role of microtubule-dependent vesicle trafficking for bristle construction. *BicDR* supports the function of *BicD*, and we discuss the hypothesis whereby BicDR might transport cargo more locally, with BicD transporting cargo over long distances, such as to the distal tip. We also identified embryonic proteins that interact with BicDR and appear to be BicDR cargo. For one of them, EF1γ (also known as eEF1γ), we show that the encoding gene *EF1γ* interacts with *BicD* and *BicDR* in the construction of the bristles.

## Teaser

**Summary:** *Drosophila BicD* and *BicDR* are needed for the elaboration of the F-actin bundles of macrochaetae and proper Rab6 and Spn-F expression. BicDR appears to support BicD in vesicle transport.

## INTRODUCTION

Microtubules are crucial for the growth of polarized cells. They also localize different cellular organelles, such as the nucleus, vesicles, the Golgi and the endoplasmic reticulum, to specific cellular compartments and enable the polarized transport of vesicles, mitochondria, mRNAs and cytoskeletal elements (Bartolini and Gundersen, 2006; Bitan et al., 2010a,b). Because of their growth, which is focused towards one pole of the cell (Bitan et al., 2010a), the *Drosophila* macrochaetae can serve as a model tissue for studying such cytoskeleton-dependent transport processes, as they are necessary for bristle development (Melkov et al., 2016). Several studies indicate that vesicle trafficking has an important function in this process (Rodriguez-Boulan et al., 2005). Multiple defects in bristle development have been described in flies that are mutant for members of the *Rab* gene family, which is known to regulate intracellular vesicle trafficking. Whereas *Rab6* and *Rab11* mutants eclose with short and stubble-like bristles, *Rab35* mutants display forks and kinks in their macrochaetae (Nagaraj and Adler, 2012; Purcell and Artavanis-Tsakonas, 1999; Zhang et al., 2009, 2007).

*Drosophila* Bicaudal-D (BicD) is part of an evolutionarily conserved transport machinery, the microtubule-dependent dynein–dynactin transport apparatus. Its essential functions in the development of the oocyte and embryo are well characterized (Claußen and Suter, 2005; Schlager et al., 2010; Vazquez-Pianzola et al., 2022, 2014). Furthermore, it has been observed that *BicD^A40V, S103A^* and *BicD^null^* mutants display short sternopleural and scutellar bristles (Koch et al., 2009). This mutant phenotype pointed to a function of *BicD* in the development of macrochaetae, one that had not been studied so far. The similarity between the bristle phenotype of *BicD* and *Rab6* suggested possible interactions between these two in constructing the bristles. Support for this hypothesis also comes from work that showed that *Rab6* and *BicD* function together in the delivery of secretory pathway components (Januschke et al., 2007).

The BicD protein family contains another member, BicD-related (BicDR, encoded by *CG32137* in flies), which was discovered due to its strong sequence similarity to BicD. In *Danio rerio*, *BicDR* is needed for the pericentrosomal transport of Rab6-positive vesicles during neural development. To perform this function, the *Danio rerio* BicDR requires the lysine residue K512, which is highly conserved between BicD and BicDR paralogs and orthologs Schlager et al., 2010). The *Drosophila* BicD sequence around K730 is essential for the interaction with the cargo (Dienstbier et al., 2009) and a point mutation in this codon resulted in the isolation of the first single amino acid substitution that produced a *BicD^null^* phenotype, indicating that this lysine is key to the physiological role of BicD (Ran et al., 1994). The homologous lysine in *Drosophila* BicDR is conserved and located at position K555 of the BicDR-B isoform and K461 of the BicDR-A isoform. For simplicity, we will refer to this residue as K555 for both isoforms. However, whether K555 of *Drosophila* BicDR serves the same function as its K730 ortholog of BicD remains to be tested. There are also interesting differences between BicD and BicDR. Whereas *Drosophila* BicD consists of three coiled-coil domains, only the first and third are conserved in the BicDR protein.

Although the strong similarity between the fly BicD and BicDR suggests similar functions, the role of *BicDR* in *Drosophila* has not yet been examined. The similarity is strongest in the coiled-coil domain near the C-terminus which, in the case of BicD, is known to be needed for the attachment and transport of various cargoes (Dienstbier et al., 2009; Schlager et al., 2010).

We set out to investigate the function of *BicDR* with a focus on a potential role in MT-dependent trafficking and possible cooperation or competition between BicDR and BicD that might contribute to the development and maintenance of polarized cell growth. Here, we describe the genetic interaction between *BicD* and *BicDR* and its contribution to fly development. Furthermore, we describe and compare the effect of different *BicD* and *BicDR* alleles on the formation of the macrochaetae.

## RESULTS

### Functional redundancy between *BicDR* and *BicD*

Whereas BicD consists of three coiled-coil domains, only the first and third are conserved in the related BicDR protein (Fig. S1A). The similarity is strongest in the coiled-coil domain near the C-terminus, which in the case of BicD, is known to be needed for the transport of different cargoes (Dienstbier et al., 2009; Schlager et al., 2010). Although the N-terminus is less highly conserved between the two proteins, it has been shown that BicD and BicDR require it for the efficient binding to dynein and dynactin *in vitro* (Splinter et al., 2012; Urnavicius et al., 2018).

*Drosophila BicDR* extends over 24.4 kb of genomic sequence with 6–8 exons in total (Fig. S1B). The relatively long intron with ∼18 kb between the first and second protein-coding exons is remarkable, and this structure is similar to the structure of the *BicD* gene (Thurmond et al., 2019). There are two transcripts, *BicDR-A* and *BicDR-B*, with the difference that the *BicDR-A* start codon is localized 282 bp downstream of the *BicDR-B* one. Both transcripts share the reading frame and the stop codon. The extra peptide of BicDR-B contains a repeat of five asparagine and six serine residues, respectively, but shows no similarity to any other gene or organism.

To identify specific *BicDR* alleles that could be null alleles, we picked two P-element insertions and created imprecise excision mutations (see the Materials and Methods section). For further genetic analyses, we retained two excision lines from the upstream element and one from the downstream insert: *BicDR^29^*, *BicDR^51^*, and *BicDR^71^* (Fig. S1B)*.* Although excision *BicDR^29^* removed only the 5′ UTR region, excision *BicDR^51^* also removed, in addition to that, the entire first protein-coding exon. *BicDR^71^* is the only excision that removes the second, third and fourth protein-coding exon and thereby also induces a stop codon in the first coiled-coil domain of BicDR-A and BicDR-B (Fig. S1). In addition, we retained a precise excision *BicDR^(rev)^* from the downstream element as a control. We also generated a precise mutation where the Q554 and K555 codons in *BicDR* were deleted (*BicDR^8.1^*, see Fig. S1B).

**Fig. 1.**
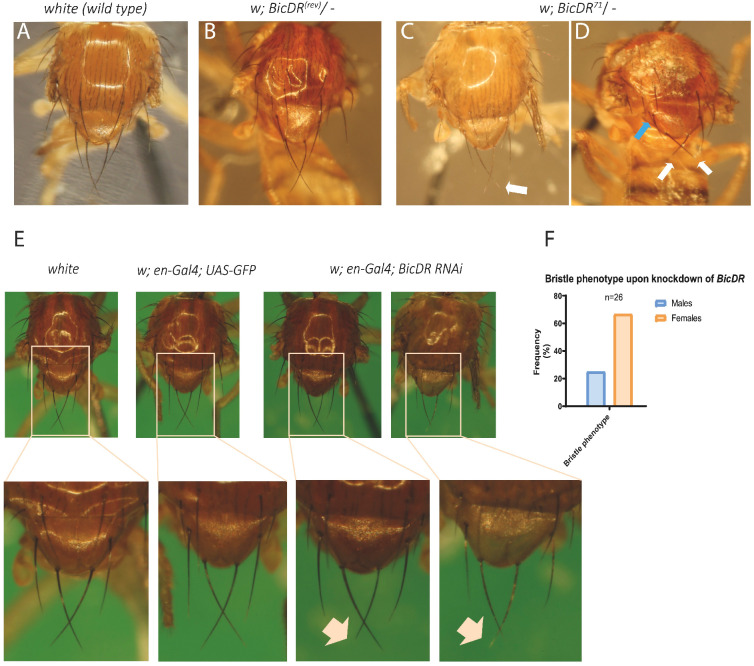
**Bristle phenotypes in *BicDR* mutants.** (A) Macrochaetae of a wild-type control fly, the revertant *w*; *BicDR^(rev)^*/*Df4515* (B) and *w*; *BicDR^71^*/*Df4515* (C,D). Note that the notum of the *w*; *BicDR^(rev)^*/*Df4515* control does not show any differences compared to the wild type, whereas *w*; *BicDR^71^*/*Df4515* flies eclosed with shorter pSC macrochaetae (white arrows in D) and occasionally with an additional aSC bristle (blue arrow in D); identification of macrochaetae was according to Takano (1998). Arrow in C indicates a discolored bristle tip. Note that several of the mutant phenotype images are reproduced in Fig. 6 for ease of comparison. Images in A–D representative of 20–40 animals examined. (E) RNAi knockdown of *BicDR* induces defective bristles. *w*; *UAS-BicDR-RNAi*/*en-Gal4*; *UAS-BicDR-RNAi*/*UAS-GFP*. Note the slightly paler macrochaetae tips and occasional shorter macrochaetae (marked with an arrow). In A–E, thorax lengths were 1.0–1.1 mm. (F) More female flies displayed the bristle phenotype upon knockdown of *BicDR* (12 out of 18 female and 2 out of 8 male flies).

All the described *BicDR* mutants were viable and fertile, indicating that *BicDR* is a non-essential gene. However, hemizygous *BicDR^71^* females eclosed with individual macrochaetae that contained discolored and brittle tips that bent or broke off easily (Fig. 1; white arrow pointing to a discolored bristle tip in Fig. 1C). Additionally, a fraction of the female adults contained additional aSC, aPA or pNP macrochaetae (Takano, 1998) (see white arrows pointing to shorter pSCs and a blue arrow to an additional aSC in Fig. 1D). This phenotype was not observed in *BicDR^29^* mutants. Knocking down *BicDR* by RNAi driven with the *en-Gal4* driver also led to adult females eclosing with individual slightly shorter aSC or pSC macrochaetae (Fig. 1E). This phenotype could be observed significantly more often in females than in males. 12 out of 18 female and 2 out of 8 male flies eclosed with at least one shorter bristle, whereas no control animals (0 out of 32 *en-Gal4; UAS-GFP*) displayed such a phenotype (Fig. 1F). These results show that *BicDR* functions in the formation and development of mechanosensory organs of *Drosophila* during metamorphosis.

The sequence similarity between BicD and BicDR suggests that the two proteins might either be functionally redundant or compete with one another. To test these possibilities, we produced flies that simultaneously carried mutations in both genes using a female sterile allele of *BicD* (*BicD^PA66^*) (Schüpbach and Wieschaus, 1991; Suter and Steward, 1991). *BicD*; *BicDR* double mutants of the genotype *BicD^PA66^*/*−*; BicDR*/*−* were tested for viability and fertility. BicDR* stands for the different *BicDR* alleles tested (*BicDR^29^*, *BicDR^51^*, *BicDR^71^* and *BicDR^8.1^*) and the wild-type revertant *BicD^(rev)^* that served as a control.

**Fig. 2.**
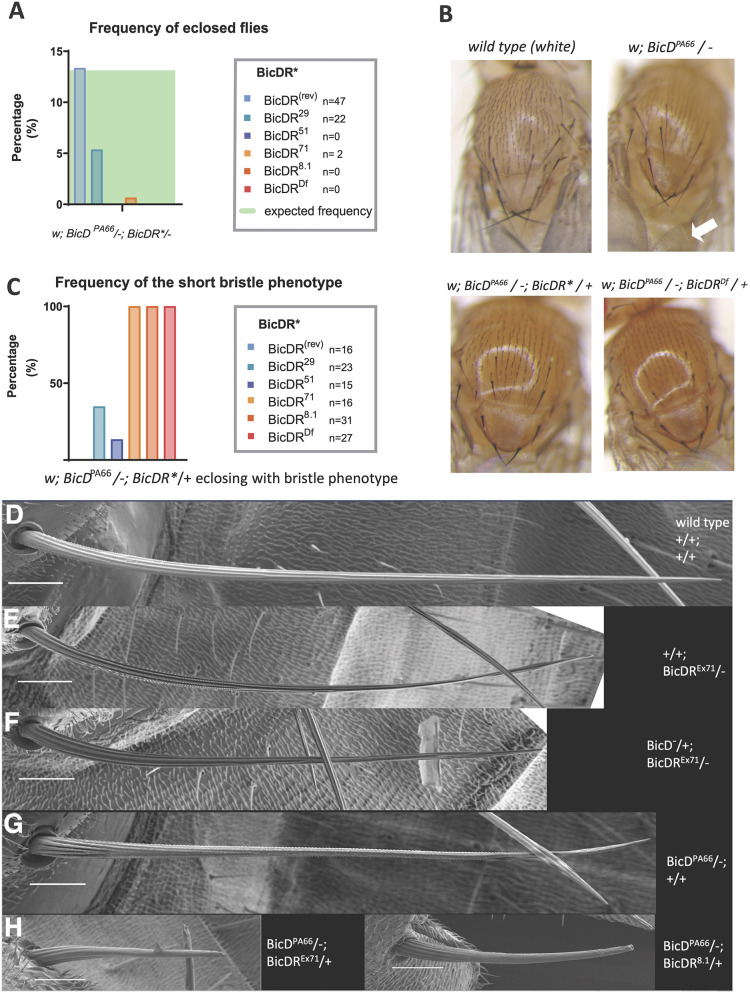
**Effect of *BicD* and *BicDR* mutations on the structure of the macrochaetae.** (A) Frequency of eclosed *BicD*; *BicDR* double mutants with the genotype *w*; *BicD^PA66^*/−; BicDR*/−. Adult flies eclosed only in crosses containing *BicDR^29^* or the control *BicDR^(rev)^* except for one escaper with the allele *BicDR^71^*. All of them developed a short-bristle phenotype. The genotype *w*; *BicD^PA66^*/−; *Df4515*/+ eclosed in all crosses and all flies with the named genotype developed the short-bristle phenotype as well. The calculated expected frequency is shown in green. (B) Comparison of the bristle phenotypes observed in controls [*white* (*w*)], *BicD^PA66^/−* and *BicD^PA66^/−*; *BicDR^71^/+* as well as *BicD^PA66^/−*; *BicDR^Df^/+*. Note that the *BicD* bristle phenotype, which manifests itself in discolored and brittle bristle tips, is stronger upon the reduction of *BicDR* function. The phenotypes observed in *BicD^PA66^/−*; *BicDR^71^/+* and *BicD^PA66^/−*; *BicDR^Df^/+* show the same severity, indicating that the allele *BicDR^71^* behaves like a *BicDR^null^* mutant for this phenotype. Arrow indicates a discolored bristle tip. Thorax lengths were 1.0–1.1 mm. (C) The frequency of the short bristle phenotype in *BicD^PA66^*/−; BicDR*/+ animals. The *BicDR*-excisions that the animals carry and the deficiency *Df737* (this deficiency is also referred to as *BicDR^Df^*), are indicated. (D–H) Scanning electron micrographs of the posterior scutellar bristles (pSC) of the wild type, *BicD* and *BicDR* mutants and mutant combinations, showing their effects on bristle length and structure. Scale bars: 30 μm. (D) Wild type is an OreR line outcrossed to a *white* line. (E) *BicDR^−^* mutants show slightly shorter, thinner bristles that appear flattened. (F) With only one normal copy of *BicD*, *BicDR* bristles are again slightly shorter. (G) *BicD^PA66^/−* bristles are only slightly shorter than wild-type bristles, but the enhancement of the phenotype by inactivating one copy of *BicDR* – shown with two unrelated alleles – is very strong (H). Note that in this genotype, the bundle structure is only visible close to the base and gets lost 50 μm distal to the base. ‘-’ indicates that the *BicD* or *BicDR* gene on the indicated chromosome was deleted by a small deficiency (Df7068 for *BicD* and Df4514 for *BicDR*). Image in D is representative of eight wild-type bristles. Image in E is representative of 16 bristles of the genotype *BicDR*^*71*^ over deficiency or *BicDR*^*8.1*^. Image in F is representative of 16 bristles of the genotype *BicDR*^*71*^ over deficiency or *BicDR*^*8.1*^. Image in G is representative of ten bristles of the genotype *BicDR*^*PA66*^ over deficiency. Images in H are representative of 12 (left) and eight (right) bristles.

As shown in Fig. 2A, hemizygous double mutant males and females were virtually absent from the offspring but appeared in the control *BicD^PA66^*/−; *BicDR^(rev)^*/− (13% of the total number of eclosed progeny, which is the expected frequency for the control). The genotype *BicD^PA66^*/−; *BicDR^71^*/− was only found in three male flies (0.65% of all eclosed progeny; Fig. 2A). No progeny of the genotypes *BicD^PA66^*/−; *BicDR^51^*/− and *BicD^PA66^*/−; *BicDR^Df^*/− or *BicD^PA66^*/−; *BicDR^8.1^*/− eclosed, whereas 21 animals with the genotype *BicD^PA66^*/−; *BicDR^29^*/− eclosed (5.37% of the total eclosed progeny). This shows that the genotype *BicD^PA66^*/− is viable, but if both copies of *BicDR* are additionally null or strong loss-of-function alleles, the flies are not viable anymore. We conclude that one functional copy of *BicDR* is sufficient to support the residual *BicD* function in *BicD^PA66^/−* and maintain viability*.* This points to a redundant role of *BicD* and *BicDR* for an essential function.

The few *BicD^null^* animals that survived to adulthood displayed a bristle defect phenotype with colorless and brittle bristle tips (Koch et al., 2009). Whereas the discolored tips were also seen in *BicD^PA66^*/− flies (Fig. 2B), much shorter bristles only appeared when *BicDR* activity was also reduced in this background (*BicD^PA66^*/−; BicDR*/+). These animals eclosed with significantly shorter, stubble-like macrochaetae (Fig. 2B). This short bristle phenotype was the strongest in flies that carried the *BicDR^71^* and *BicDR^8.1^* allele; all adult progeny with a hemizygous copy of *BicD^PA66^* and one *BicDR^71 (or 8.1)^*/+ chromosome showed the short bristle phenotype (Fig. 2C). The same was true when the *BicDR* deficiency chromosome (*BicDR^Df^*) was tested in the same way (*BicD^PA66^*/−; *BicDR^Df^*/+). In contrast, less than half of the hemizygous *BicD^PA66^* flies containing *BicDR^51^* or *BicDR^29^* eclosed with short bristles. For *BicDR^29^*, these were 35% (8 out of 23 flies) and for *BicDR^51^* 33% (5 out of 15 flies). These results show again that *BicDR^+^* supports *BicD^PA66^* in bristle development but only a single functional copy of *BicDR* is not sufficient to allow normal bristle development.

The genetic analyses of the mutant *BicDR* alleles define an allelic series. The *BicDR^29^* is a hypomorphic allele and produces the weakest phenotype because hemizygous *BicD^PA66^* animals that are also hemizygous for *BicDR^29^* are viable, whereas the analogous genotype is lethal for *BicDR^71^*, *BicDR^51^*, or *BicDR^Df^*. *BicDR^29^* seems to retain considerable functional *BicDR* activity, and this seems possible because the excision only removed the 5′ UTR region and intron sequences of *BicDR* but no protein-coding regions (Fig. S1). By contrast, the *BicDR^71^* allele is the strongest. Our results reveal that the allele *BicDR^71^* induces the strongest effect within flies that contain the hypomorphic mutation *BicD^PA66^*. We can further conclude that *BicDR^71^* is a stronger *BicDR* allele than *BicDR^51^* and that its behavior can be compared to the deficiency of *BicDR*, *BicDR^Df^*, which removes the *BicDR* gene completely. *BicDR^71^* removes protein-coding exons 2 and 3, whereas *BicDR^51^* removes the 5′ UTR region and the first protein-coding exon of *BicDR-A* and *-B* (Fig. S1). The independently generated allele *BicDR^8.1^* (equivalent to *BicDR^ΔGK555^*) also appeared to be a null allele like *BicDR^71^*, but was not tested as much as the latter.

A more detailed picture of the different bristle phenotypes was obtained with by scanning electron microscopy (SEM; Fig. 2D–H; Fig. S2). The average length of unbroken pSC bristles was ∼455 µm for the wild type (Fig. 2D). The *BicDR*/− pSCs reached 82% of this length (Fig. 2E; the average of the measured length was 375 µm) and the BicD*/+; *BicDR*/− pSCs reached 70% of the normal size (Fig. 2F; 320 µm; BicD* stands for *BicD^PA66^* or the *BicD* deficiency). *BicD^PA66^*/− pSCs reached 78% of the normal size (Fig. 2G; 355 µm) and additional inactivation of one copy of *BicDR* brought this down to only 35% of the normal size (Fig. 2H; 160 µm). Bristles contain actin filament bundles, which are known to shape the cuticle ridges seen on the surface of the adult bristles. These ridges are prominently seen in the proximal region close to the base where they are separated by deep grooves (Fig. 2D). In the wild-type bristles, these ridges (and presumably also the underlying actin bundles that shape them) are amazingly straight all the way to the region of the tip, and only a few ridges seem to merge. In apparently full-length *BicDR* bristles, the ridges are also seen in the tip, but the tips often appear frayed with individual ridges or small groups of ridges separating from others, dissolving the tip into several small tips (Fig. S2). This suggests that the underlying actin bundles are less ‘glued together’ and less straight. In the *BicD^PA66^*/− situation, the ridges are straight in most parts of the bristle, but the grooves, which seem to form normally close to the base, become less prominent in the more-distal regions. In the still conical tip, the ridges with the bundle structure appear less straight and more twisted or braided. This phenotype becomes strongly enhanced by removing one copy of *BicDR*. In the *BicD^PA66^*/−; *BicDR^−^*/+ background, the ridges with their bundle structure are apparent only very close to the base and get lost on the surface in the more-distal region (see also Fig. 2H). This phenotype closely resembles the *Rab6/warthog* phenotype described by Purcell and Artavanis-Tsakonas (see Fig. 2d in Purcell and Artavanis-Tsakonas, 1999). In this background, we observed frequently broken bristles with a large diameter in proximal bristle regions (Fig. S2).

In the *BicDR* mutants, we observed very thin and flattened bristles in the more-distal regions and many displayed kinks and frayed tips. If these mutants have only one *BicD^+^* copy, the tips appear more twisted and braided (Fig. S2).

Lack of *BicDR* function also led to a slightly reduced bristle thickness in the proximal region just above the bristle base (9–10 µm compared to slightly over 10 µm; overview pictures in Fig. S2). Surprisingly, the phenotype of the *BicD^PA66^*/− bristles differed from the wild-type in the opposite direction. These bristles were between 11 and 13 µm thick in the corresponding proximal region.

One mechanism by which BicDR might support BicD function is suggested by their similar structure. BicD functions as a dimer, which it forms through its coiled-coil domains. If the homologous coiled-coil domains interact, BicDR might replace a BicD subunit in the active complex. To test whether BicD and BicDR form heterodimers we tested for this interaction in a yeast two-hybrid experiment, which would reveal direct interactions between the two proteins. The yeast two-hybrid experiment confirmed that BicDR forms homodimers (Fig. S3), as had been already described (Chaaban and Carter, 2022; Urnavicius et al., 2018). However, the experiment did not reveal any direct interaction between BicD and BicDR. Similarly, immunoprecipitations (IPs) with embryos expressing GFP-tagged BicDR did not reveal copurification of the two related proteins by western blotting or mass spectrometry (MS) analysis (see below). Therefore, our results do not provide evidence that BicDR supports BicD function by forming dimers.

### BicDR::GFP is expressed in the salivary glands and the embryo in a metameric pattern

**Fig. 3.**
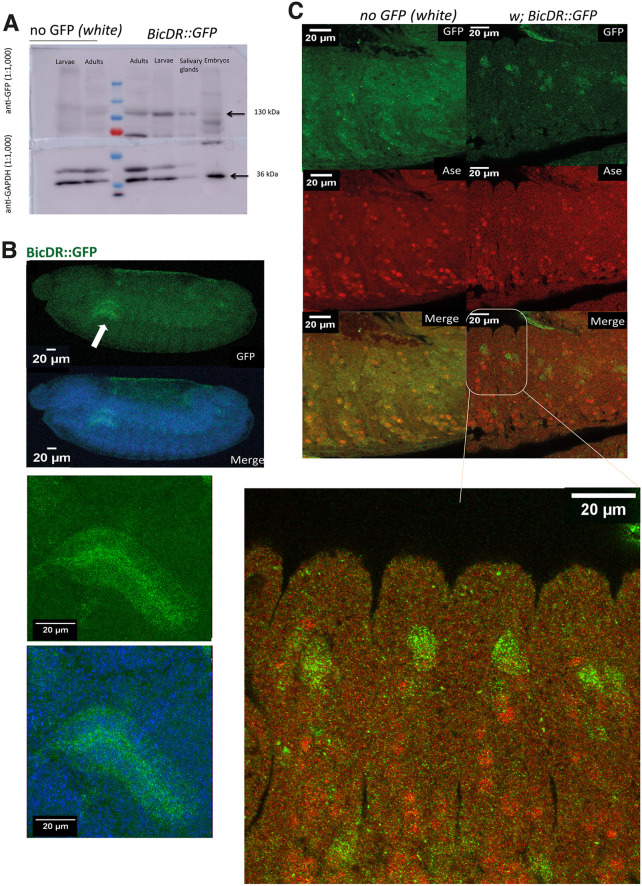
**BicDR is expressed in the region of sensory organ precursors of stage 13 embryos.** (A) Expression during the different stages of the life cycle is shown by a western blot stained for GFP to reveal the expression of the endogenously tagged *BicDR* (see Fig. S6 for uncropped images of the blot). The samples loaded were from stage 13 to 16 embryos, third-instar larvae, adult flies and salivary glands. All were of the genotype *w*; *BicDR::GFP*/*Df4515* or the negative control (*white*). The loading control was GAPDH with a size of 35 kDa (see lower blot). BicDR-B::GFP, with a size of 130 kDa was found mostly in adult flies, 3rd instar larvae, and the dissected salivary glands of the third-instar larvae, while BicDR-A::GFP with 110 kDa was mostly expressed in late embryos. Blot is representative of two repeats. (B) Stage 13 embryos stained for BicDR::GFP (green). The DNA is stained with Hoechst (blue). BicDR::GFP is expressed apically in the cells of salivary glands and cells along the anterior-posterior embryo axis in a metameric pattern. Arrow highlights salivary gland. Magnified views are shown underneath. (C) Co-staining of BicDR::GFP embryos with the sensory organ precursor marker Asense (red) and GFP (green) identifies the GFP-positive cells in the vicinity of elevated Ase staining. Images in B and C are representative of five (B) and seven (C) repeats.

To determine in what tissues and during which embryonic stages BicDR is expressed, we tagged the *BicDR* gene endogenously with GFP using CRISPR/Cas9 and immunolocalized BicDR::GFP in embryos after fixation. This method allows us to track both translated BicDR-A and BicDR-B. Through sequencing, we confirmed that the BicDR open-reading frame (ORF) was fused seamlessly with the eGFP ORF. The successful ORF fusion and the expression of the predicted fusion protein were also confirmed by western blotting of embryonic extracts, which revealed the GFP expression as part of a 130 kDa polypeptide (Fig. 3A).

Immunolocalization of BicDR::GFP in embryos revealed that the apical side of salivary gland cells stained very strongly from stage 13 on (white arrow in Fig. 3B, higher magnification shown underneath). Additionally, individual cells displayed staining signals in a metameric manner along the lateral side of the embryo. These signals were most intense during stages 11–14 (Fig. 3C, top). Co-immunostaining of the cytoplasmic BicDR::GFP and the nuclear neuroblast marker Asense (Ase), which is expressed in all sensory organ precursor (SOP) cells and their progeny (Berdnik et al., 2002), reveled that the cells with the strongest BicDR::GFP signal are seen in the region of Ase-positive cells. Although some stronger signals seem to overlap, often the two different signal peaks appeared to be in adjacent cells (Fig. 3C). Because initial experiments did not reveal a function of *BicDR* in salivary glands but identified defects in the adult bristles of the mutants, we focused on the function of *BicDR* in bristle development.

### *BicD* and *BicDR* contribute to localizing Rab6 to the tip of the mechanosensory bristles

**Fig. 4.**
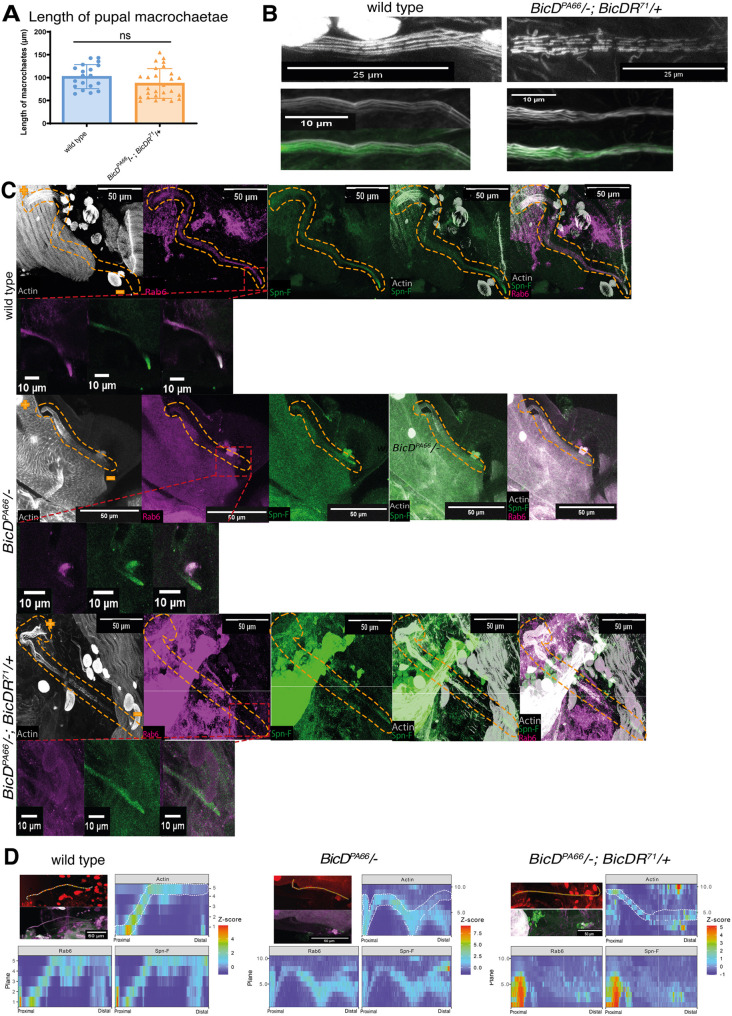
**Pupal bristles depend on *BicD* and *BicDR* for actin bundle stability and proper localization of Spn-F and Rab6.** (A) The length of single pupal macrochaetae was measured in *white* controls and *BicD^PA66^*/−; *BicDR^71^*/+ double mutants. No significant dissimilarities (ns) between the two groups were found at this stage of bristle development (two-tailed *t*-test). (B) Scutellar macrochaetae stained for F-actin and acetylated tubulin in controls and the indicated double mutant (gray, F-actin; green, acetylated tubulin). Images are representative of four (wild type) and seven (double mutant) repeats. (C) *BicD* and *BicDR* are needed to localize normal levels of Rab6 in the shaft of scutellar macrochaetae and at their bristle tips. This accumulation is impaired in the *BicD^PA66^/−* and particularly in the *BicD^PA66^*/−; *BicDR^71^*/+ double mutants (gray, F-actin; green, Spn-F; pink, Rab6; see also D and Figs S4, S5 for additional staining and relative quantification). The genotype of the sample is listed on the left side. All macrochaetae originate in the upper left corner (indicated with a ‘+’) and grow downwards to the lower right corner (indicated with a ‘–’) and are highlighted by orange dashed lines. The tip is visualized with staining for Spn-F. The enlargement of the bristle tips framed by the red boxes in C is shown for the three channels. The outlines of the bristle cells were estimated from the F-actin staining and the staining for Spn-F and Rab6. The localization of Rab6 decreases toward the bristle tip of *BicD* mutants, whereas it was completely absent in the distal tips of the *BicD*; *BicDR* double mutants. (D) Intensity plots of Rab6 and Spn-F signals in each image plane to visualize the distribution of Rab6 and Spn-F signals through the bristle shaft. The highest *Z*-score of 4 is shown in red; dark blue marks a *Z*-score of 0 and indicates that no signal could be detected. The segmented line drawn through the bristle shaft has a width of 10 pixels and their mean result was used for the graphs. Images in D are representative examples for five repeats each.

To further understand bristle development and the impact of *BicD* and *BicDR* on it, pupal dorsal tissue containing the developing bristles was dissected 40 to 44 h after pupation, fixed and stained. In this way, hemizygous *BicD^PA66^* samples with only one functional copy of *BicDR* were compared to *BicD^PA66^* animals with two functional *BicDR* copies and to controls that were wild type for *BicD* and *BicDR*. Investigating the F-actin structure of the samples allowed us to compare the length and morphology of the macrochaetae (Fig. 4A,B). Comparing the pupal bristle length in the mutants with the wild type showed that the mutant bristles appeared to be somewhat shorter but there were no significant length differences (Fig. 4A). This indicates that the short bristle phenotype observed in *BicD*; *BicDR* double mutant flies occurred at a later stage of development. Although similar in length, the actin cytoskeleton of the double mutant scutellar bristles displayed abnormalities; 6 out of 7 mutant scutellar macrochaetae showed an irregular arrangement of the actin bundles, and obvious gaps could be observed (Fig. 4B). This phenotype was not observed in the control pupae (0 out of 4 scutellar macrochaetae) nor in hemizygous *BicD^PA66^* animals. Although such gaps in the actin bundles are reminiscent of what occurs during the chitinization process for the bristles (Tilney et al., 1996), chitinization does not appear to be the reason for the phenotype, because all pupae were only 40 to 44 h into pupation and the breakdown of the bundles by chitinization begins only 48 h after pupation. Chitinization also initially causes narrow longitudinal gaps between modules, and these become wider as the bristle ages, with breakdown only becoming clearly recognizable in 53-h-old pupae (Tilney et al., 1996).

Rab6 is known to be a Notch modifier that influences the development of the mechanosensory bristles on the head, notum and scutellum. The *Rab6* phenotype also results in aberrant bristle length and bristle tips that have very mild and disorganized ruffling (Purcell and Artavanis-Tsakonas, 1999). This phenotype resembles the short bristle phenotype observed in *BicD*; *BicDR* double mutants. Additionally, Schlager and colleagues have described a physical interaction between Rab6 and BicDR in *Danio rerio* (Schlager et al., 2010), and Januschke et al. an interaction between *Drosophila* Rab6 and BicD (Januschke et al., 2007). We, therefore, examined the Rab6 distribution in the macrochaetae of *BicD^PA66^/−* and *BicD^PA66^*/−; BicDR*/+ mutants. For this, we stained the pupal dorsal tissue for Rab6. As seen in Fig. 4C,D and Fig. S4, in the wild-type scutellar bristle the Rab6 signal is present along the entire shaft, but there appears to be a higher Rab6 level at the proximal end and a gradual reduction towards the distal end, often followed by a second, smaller peak at the tip. For Fig. 4D and Fig. S4, channel signal intensities were normalized per channel, allowing a better impression of the signal distribution in the bristle. Fig. S5 shows the same primary data normalized across all three channels. This allows one to detect changes in signal levels from genotype to genotype if the experimental conditions are the same and the background signals are low. With the settings used to image the pupal bristles, the Rab6 signal in the wild type had an intensity similar to that of the F-actin signal. In the *BicD* mutants and particularly in the double mutants, Rab6 levels were drastically reduced compared to the F-actin signal and only weakly seen in parts of the bristle (Fig. S5), revealing that Rab6 expression levels strongly depend on functional *BicD* and *BicDR*. In *BicD^PA66^/−*; *BicDR/−* mutants, residual Rab6 signal appears to be evenly distributed throughout the bristle shaft without a discernable distal tip accumulation.

### *BicDR* and *BicD^PA66^* in localizing Spn-F to bristle tips

**Fig. 5.**
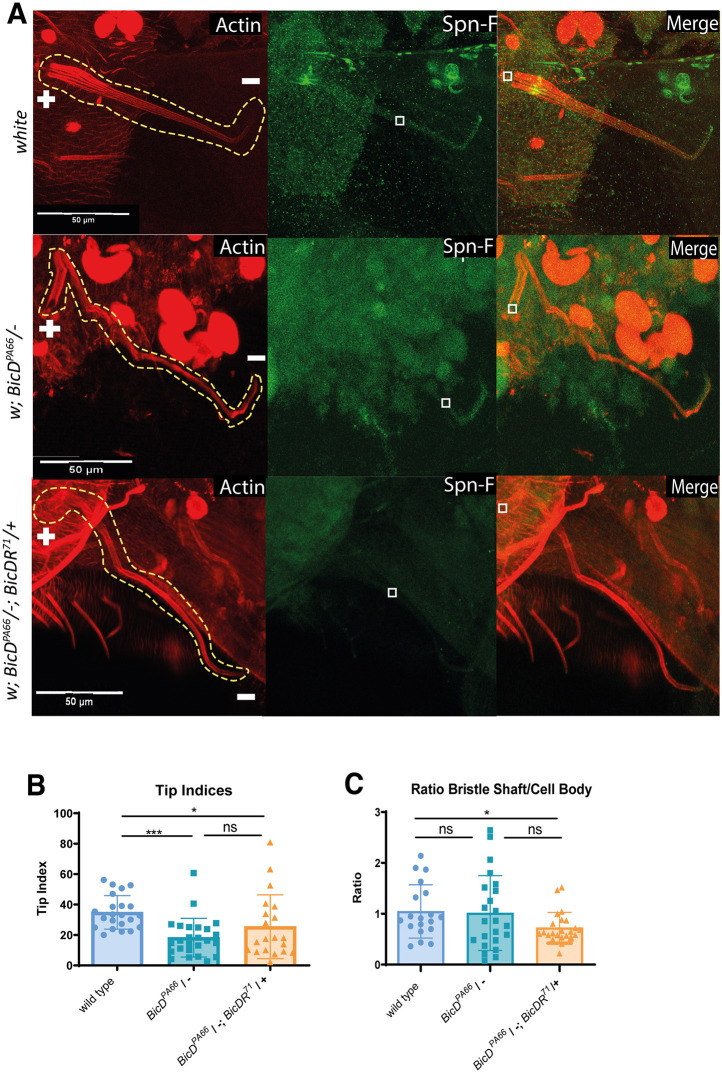
**Pupal bristles of *BicD*; *BicDR* double mutants show impaired Spn-F localization at the bristle tip.** (A) Stained macrochaetae (red, F-actin; green, Spn-F). The genotypes of the samples are listed on the left side. All macrochaetae originate in the upper left corner and have their tips pointing downwards (bristles are outlined by the dashed yellow line, + is the proximal end, − the distal end). The localization of Spn-F at the bristle tip is much weaker in *w*; *BicD^PA66^*/*Df7068*; TM6B/+ and *w*; *BicD^PA66^*/*Df7068*; *BicDR^71^*/+ pupae. This observation could be confirmed with the calculation of the tip index shown in B. (B) The average tip index of the mutants is significantly lower than those of the control group. (C) The ratio of Spn-F signal in the elongated bristle shaft versus its cell body is significantly lower in *BicD^PA66^*/−; BicDR*/+ animals. White squares indicate the positions within the bristle shaft where the signal intensity of Spn-F was measured. For the calculation, the signal was measured within one plane in the approximately middle part of the bristle shaft and divided through the signal intensity measured within the plane where the actin bundles sprout out of the tissue. Error bars are s.d. In B and C, *n*=19 (wild type), 23 (*BicD^PA66^/–*) and 24 (*BicD^PA66^/–*; *BicDR^71^/+*). *P<0.05; ***P<0.001; ns, not significant (one-way ANOVA with Dunnett's multiple comparisons test).

Spindle-F (Spn-F) is a microtubule minus-end marker that affects oocyte patterning and bristle morphology in *Drosophila* (Abdu et al., 2006). *Spn-F* mutants eclose with shorter and thicker bristles. Scanning electron micrographs of the bristles revealed that the mutant bristles have branching tips and that the direction of elongation is sometimes perturbed (Abdu et al., 2006). Spn-F functions at the distal tip of the growing bristle and is involved in the regulation of the shuttling movement of recycling endosomes and cytoskeletal organization (Otani et al., 2015). We analyzed the potential requirement for *BicD* and *BicDR* for the localization of Spn-F to and within the shaft of the bristle cells (Figs 4C, 5; Figs S4, S5). The normal asymmetric localization to the tip of the macrochaetae allowed us to assess the contribution of *BicD* and *BicDR* to this microtubule minus-end transport process. One measure for the establishment of the polarity of the bristles is the ‘tip index’: a line scan from the bristle shaft to the distal tip establishes a plot profile from which the maximum intensity along the bristle length is determined. The ‘tip index’ is defined as the relative position of the pixels that exceed 50% intensity along the bristle axis (Otani et al., 2015). This index is used to quantify the asymmetric localization of a protein within the bristle cell. If a signal is completely localized at the bristle tip, the tip index will have a value of 100. If the signal remains in the cell body and stays absent from the bristle, the value of the tip index is 0 (Otani et al., 2015). This measurement confirmed that the Spn-F signal is significantly more concentrated at the tip of the macrochaetae of control pupae, whereas this signal tends to appear diffusely throughout the whole cell in *BicD^PA66^*/− and *BicD^PA66^*/−; *BicDR^71^* /+bristles (Fig. 5A,B). The tip index in control macrochaetae had a value of 33, whereas the value in *BicD^PA66^*/−; *BicDR^71^*/+ bristles was 18. Similar to this, the tip index in *BicD^PA66^*/− bristles was 16. These results suggest that *BicD* is necessary for the localization of Spn-F to the distal tip.

**Fig. 6.**
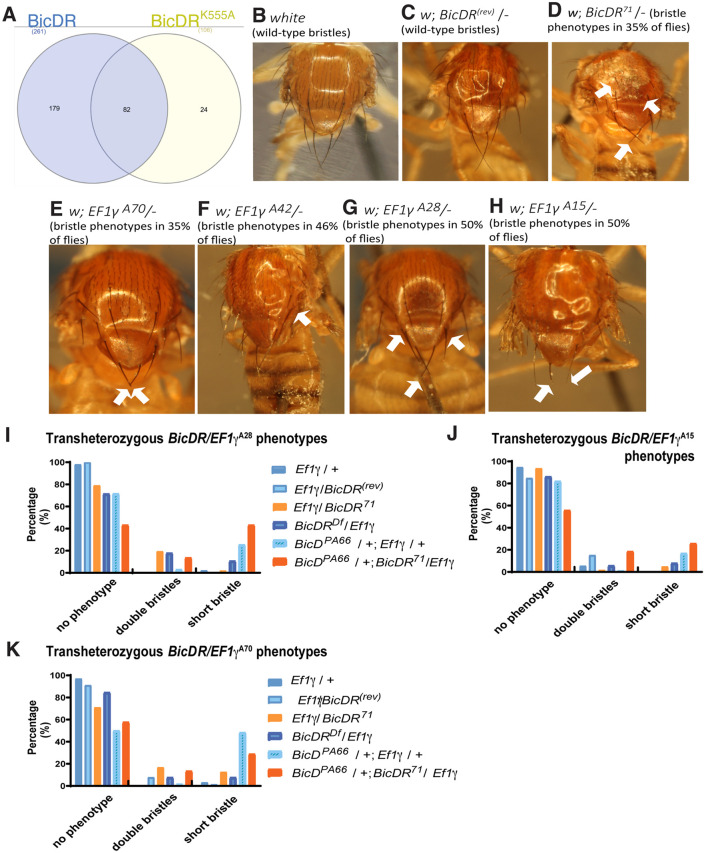
**Genetic interaction between *BicDR* and *EF1γ* in bristle construction.** (A) Proteins identified in the cut-out gel bands from the tagged BicDR and BicDR^K555A^ immunoprecipitations. A total of 285 potential binding partners were identified; 82 proteins were found in both samples, whereas 179 proteins were found only in the wild-type BicDR::GFP IP. (B–H) Resemblance of phenotypes compared to the (B) *white* control (0% short bristle phenotypes; *n*=21), (C) *BicDR^(rev)^*/− control (0% short bristle phenotype; *n*=13)*,* (D) *BicDR^71^*/− (35% of flies displayed such a bristle phenotype; *n*=11)*,* (E) *EF1γ^A70^*/− (35% of flies displayed a short bristle phenotype; *n*=9), (F) *EF1γ^A42^*/− (46% of flies eclosed with additional bristles; *n*=11), (G) *EF1γ^A28^*/− (50% of flies had additional and shorter bristles; *n*=26), (H) *EF1γ^A15^*/− (50% of flies had shorter bristles; *n*=18). Note that the *white* control and 93% of the *BicDR* revertants, *BicDR^(rev)^*/−*,* eclosed without a visible bristle phenotype. A total of 7% of *BicDR^(rev)^*/− animals contained an additional aSC bristle. Flies with the genotypes *BicDR^71^*/−, *EF1γ^A70^*/−, and *EF1γ^A51^*/− showed shorter pSC macrochaetae. Additionally, 21% of *BicDR^71^*/− animals eclosed with an extra aSC bristle, a similar frequency to that observed with the alleles *EF1γ^A42^*/− and *EF1γ^A28^*/−. Arrows point to shorter posterior macrochaetae (D,E,G and H) and to more anterior bristle duplications (D,F and G). Note that several of the mutant phenotype images shown in Fig. 1 are reproduced here for ease of comparison. Thorax lengths were 1.0–1.1 mm. Images are representative of three repeats. (I–K) Effect of combining heterozygous *EF1γ^A70^*, *EF1γ^A42^* or *EF1γ^A28^* with heterozygous *BicD* and *BicDR* alleles showing strong genetic interactions between heterozygous *BicD*, *BicDR,* and *EF1y* alleles. Frequency of mutant phenotypes observed in double and triple heterozygous combinations. Different mutant combinations containing a BicDR*, BicD^PA66^, and EF1γ* allele eclosed with different bristle phenotypes. The frequency of animals that eclosed with a short-bristle phenotype is significantly higher if the animals carry a *BicD^PA66^* and *BicDR^71^* allele except for the combination with *EF1γ^A70^* where the frequency of flies with short bristles was the highest in *BicD^PA66^*/+; *EF1γ^A70^*/+ (30%). Noticeable is that 19% of the flies with the genotype *BicD^PA66^*/+; *BicDR^71^*/*EF1γ^A28^* eclosed with short bristles, even though the allele *EF1γ^A28^* /− induces additional bristles. *n*=44–84 (I), 37–95 (J) and 52–78 (K).

Similar to the reduction seen for Rab6, the Spn-F signal in the pupal bristle was also strongly reduced, particularly in the *BicD^PA66^*/−; *BicDR^71^*/+ double mutants (Fig. S5). Additionally, the Spn-F signal ratio in the bristle shaft versus cell body changed between *BicD^PA66^*/− and *BicD^PA66^*/−; *BicDR^71^*/+ double mutants (Fig. 5C). To quantify this, we measured the average signal strength of an area on a plane in the center of the bristle shaft and divided this by the average signal strength of an area of the same size drawn on a plane through the bristle cell body, directly under the bristle root. Whereas *BicD^PA66^*/− bristles showed a wider distribution of this ratio, the ratio decreased significantly in *BicD^PA66^*/−; *BicDR^71^*/+ bristles in comparison to the wild type. One might, for instance, expect to find such a distribution if BicDR were more involved in localizing Spn-F to the periphery of the macrochaetal cell body and BicD more for the long-range transport along the bristle shaft towards the bristle tip.

At the developmental stage when we observed these localization differences, the length of the mutant macrochaetae was not yet significantly reduced (Fig. 4A). It thus appears that strongly reduced Rab6 and Spn-F levels in combination with a defective actin cytoskeleton might prevent normal bristle construction in *BicD^PA66^/−*; BicDR*/+ mutants. The reduced distal tip localization of Spn-F and Rab6 might be an additional factor contributing to the bristle phenotype.

### EF1γ is found in BicDR complexes, and *EF1γ* enhances the bristle phenotypes of *BicD* and *BicDR*

To learn more about the mechanisms through which BicDR contributes to transport processes in general, we used the C-terminally tagged endogenous *BicDR* (*BicDR::GFP*), which displays a wild-type bristle phenotype, and performed immunoprecipitations with an anti-GFP antibody using extracts from 10–16-h-old embryos. We also mutated the endogenous gene into a *BicDR^K555A^::GFP* gene and used it as a control because it might allow us to distinguish between the cargo that binds through the K555 region and other interacting partners of BicDR. A *white^−^* strain with an untagged *BicDR^+^* was used as another negative control. The search for interacting proteins was performed in two different ways. First, in triplicate experiments, embryos were lysed and immunoprecipitated with anti-GFP antibodies. A proteomic analysis was then performed directly on the precipitated fractions. Second, embryos were lysed in duplicates, immunoprecipitated as for the first method, and the resulting proteins separated by SDS-PAGE. The gel was stained with Coomassie Blue and only those bands found in the tagged *BicDR::GFP* fraction and not in the *BicDR^K555A^::GFP* samples were excised and analyzed. Gel slices from the corresponding position of the control samples were also analyzed.

The results of the first IP experiment defined 25 potential BicDR::GFP interactors with a *P*-value ≤0.05 and log_2_FC ≥1.0 (Table S5). Of these, seven were also found in BicDR^K555A^::GFP samples, indicating that these are binding partners that depend less on K555. Out of the remaining 18 candidates, different bristle phenotypes had already been described for mutant genes encoding four of the proteins found (Tou, RpS17, RpL27A and RpL12) (Casad et al., 2011; Hart et al., 1993; Vanolst et al., 2005), whereas RNA-binding activity had been observed for RpS5b (Kong et al., 2019) (Table S1). Whereas ribosomal proteins are a common contaminant in IPs, mutations in ribosomal protein genes lead to impaired bristle development and show a haploinsufficiency phenotype that is seen as evidence for a very high protein synthesis required for bristle development (Marygold et al., 2007). It is therefore also possible that BicDR interacts with ribosomes. Tou, on the other hand, is a transcription factor that activates proneural gene expression (Vanolst et al., 2005) and has also been found in a gain-of-function screen for genes that affect external sensory organs (Abdelilah-Seyfried et al., 2000). The overexpression of different *tou* alleles results in excess scutellar and dorsocentral macrochaetae (Abdelilah-Seyfried et al., 2000; Peña-Rangel et al., 2002).

Other noteworthy candidates identified in this IP are Rac1 and Morpheyus (Mey). Although identified with only a few counts, Rac1 was significantly enriched in the IP with the wild-type BicDR::GFP peptide. Rac1 has been associated with axial outgrowth (Bagley et al., 2014; Leiss et al., 2009), control of lumen size of salivary glands (Jenkins et al., 2022), and activities in tracheae, embryonic salivary glands (Chihara et al., 2003; Pirraglia et al., 2006) and bristles (see FlyBase; Jenkins et al., 2022), where overexpressing a mutant *Rac1* allele causes bristle defects. Rac1 is therefore interesting for further analysis of the *BicDR* function.

In the second approach with gel-purified bands, the larger sample size yielded 179 interacting proteins in the tagged wild-type BicDR::GFP IP that were not present in the IP of the tagged BicDR^K555A^::GFP mutant protein (Fig. 6A; Table S6). Because the *BicDR^71^* chromosome showed a bristle duplication, indicative of a problem in Notch-dependent binary cell fate acquisition (Le Bras et al., 2012), and because Notch signaling also depends heavily on cytoplasmic transport, we searched among the proteins identified in the BicDR::GFP IP for known trafficking regulators of the Notch receptor (Table S2). Origin recognition complex subunit 6, Vacuolar H^+^ ATPase subunit 68-2, Vacuolar H^+^ ATPase 26 kDa E subunit, Rumi, Par-6 and EF1γ (also known as eEF1γ) are all Notch-trafficking regulators that were absent in the control IPs but detected in the BicDR::GFP IPs. Except for *par-6*, loss-of-function mutations of all the genes for these candidate interactors result in bristle loss (Le Bras et al., 2012).

The translational regulator EF1γ appeared particularly interesting because its mutants are known to display a bristle phenotype (Fan et al., 2010), and it was immunoprecipitated at the highest amount among the identified potential binding partners of BicDR. Aside from its function in translation, EF1γ is known to negatively regulate the transport of several classes of membrane organelles along microtubules (Serpinskaya et al., 2014) and for its interaction with keratin bundles in mouse fibroblasts (Kim et al., 2007). For these reasons, we further investigated the BicDR interaction with EF1γ. To test whether the proteins might act in the same pathway, we first compared their mutant phenotypes (Fig. 6B–H). Whereas the alleles *EF1γ^A42^* and *EF1γ^A28^* induced additional aSC macrochaetae at either only one or both sides of the notum (Fig. 6F,G), the mutants *EF1γ^A70^* and *EF1γ^A15^* eclosed with shorter pSC macrochaetae, which is similar to what is seen for hemizygous *BicDR^71^* flies (compare Fig. 6E,H and 6D).

Flies transheterozygous for *BicDR^71^* and *EF1γ* were viable and 2–13% of them displayed shorter pSC or aSC macrochaetae (*BicDR^71^* and *EF1γ^A28^*, 2%; *BicDR^71^/EF1γ^A15^*, 5%; *BicDR^71^/EF1γ^A70^*:, 13%; Fig. 6I–K). This effect could not be observed in *BicDR^(rev)^*/*EF1γ^A28^* mutants or heterozygous *EF1γ^A28^* animals*.* The phenotype was significantly more prominent if the flies were transheterozygous for *BicD^PA66^* and *EF1γ^A28^* – 23% of those animals showed at least one shorter bristle. This went up to 48% with *BicD^PA66^*/+;*EF1γ^A70^*/+, whereas in *BicD^PA66^*/+; *EF1γ^A15^*/+ flies, 17% showed at least one short bristle (Fig. 6I–K). To test whether a mutant *BicDR* allele enhances the phenotype of transheterozygous *BicD^PA66^*;*EF1γ^A28^* mutants even further, we generated flies that were heterozygous for all three genes. 44% of all *BicD^PA66^*/+; *BicDR^71^/ EF1γ^A28^* eclosed with at least one shorter bristle (26% in *BicD^PA66^*/+; *BicDR^71^/ EF1γ^A15^* animals and 30% in *BicD^PA66^*/+; *BicDR^71^/ EF1γ^A70^*).

In summary, we conclude that, except for allele *EF1γ^A70^*, the proportion of animals with shorter bristles is significantly higher if they are heterozygous for all three mutants, *BicD^PA66^*, *BicDR^71^*, and *EF1γ*, indicating that all three genes are functioning in the same direction and contribute to proper macrochaetae development. This appears surprising because BicD and BicDR are components that are positively involved in microtubule-mediated transport whereas *EF1γ* negatively regulates it. The observed type of genetic interaction can be explained if *EF1γ* performs its function at the bristle tip and negatively regulates organelle transport there, allowing the organelles to perform their function at the tip. Unfortunately, the antibody localization of EF1γ did not allow us to test the distribution of EF1γ in the pupal bristles. Presumably because of the high signal levels in all tissues, one would need to use a more complex approach to test whether EF1γ can be linked more closely to *BicD* and *BicDR* activity.

Analyzing the frequency of bristle phenotypes revealed that many more females than males displayed defects, and additional bristles could be observed at low frequency in the mutants *BicD^PA66^*/+; *BicDR^71^/EF1γ*, but also in the controls (*EF1γ*/+ and *EF1γ*/*BicDR^(rev)^*). The similar bristle phenotype, the genetic interaction between *EF1γ* and *BicDR*, and the fact that BicDR::GFP and EF1γ co-precipitated posed the question of whether they interact directly. However, a yeast two-hybrid assay did not detect a direct interaction between BicD, BicDR or EF1γ (Fig. S3).

Because EF1γ was the top hit in this group and the genetic interaction assay testing for combined haploinsufficiency showed strong interactions with the *EF1γ* alleles (Fig. 6), we focused on *EF1γ* for the proof of principle in the present study. Interesting additional interactors from the same screen are Arp2 and Arp3 (Table S2). The Arp2/Arp3 complex is involved in the organization of the actin filaments (Mullins et al., 1998), a structure that is affected by the reduced *BicD*;*BicDR* function (Fig. 4).

## DISCUSSION

We found that *BicDR* is not an essential gene, but it has important functions in the development of the long bristles, the macrochaetae (Figs 1, 2). Additionally, one functional *BicDR* copy is essential for viability in a hypomorphic *BicD* background. In these animals with reduced *BicD* activity and only one functional copy of *BicDR*, the remaining combined activities of *BicD* and *BicDR* are not sufficient to develop bristles properly (Fig. 2). Here we showed that the reduced activity of *BicD* and *BicDR* affects the Rab6 and Spn-F levels and localization in the growing bristle, linking BicD and BicDR to the dynein-dependent microtubule transport of vesicles and bristle factors to their proper position in the bristle where they perform their function.

A different defect in the development of the bristle, the formation of a twin bristle on the notum, was seen in 21% of hemizygous *BicDR^71^* flies (Fig. 1D). This was mostly an additional aSC bristle with a hair and socket of its own. This hinted at a failed cell fate acquisition after the division of pI cells, which can result from gain-of-Notch signaling in the cell divisions leading to the sensory organ formation (Le Bras et al., 2012). This connection was also attractive because the Notch trafficking regulator EF1γ (Le Bras et al., 2012) was a top hit for BicDR-interacting proteins and transheterozygous *EF1γ*/*BicDR^Df^* flies also showed bristle duplications. *BicDR^Df^* lacks, aside from BicDR, eight other genes. However, the evaluation of the cause of this phenotype became too challenging for the present study because animals in the control group *BicDR^(rev)^*/−, a wild-type revertant generated by hopping out the P-element insertion that was used to generate the *BicDR^71^* allele through an imprecise excision, also showed twin bristles in 7% of the animals. On the other hand, excision mutants and revertants that were generated with the P-element that had inserted in the 5′ region of *BicDR* (Fig. S1) did not show this phenotype. A possible interpretation might be that the P-element chromosome had acquired a second hit that supports bristle duplications. Such second-site hits are common and often caused by local transpositions, which would explain why the *BicDR^Df^* chromosome also showed this interaction with *EF1γ* (Fig. 6I–K). Because of the difficulty of resolving this issue, we focused on the bristle growth phenotype to gain more insights into the function of *BicDR*.

The second coiled coil domain of BicD ensures that the adaptor protein remains inactive if no cargo is bound. For this, the cargo-binding third coiled coil domain folds back onto the second coiled coil, thereby blocking the dynein interaction site (reviewed by Suter, 2018). This mechanism ensures that the BicD-containing transport machinery does not run unloaded along microtubules. BicDR lacks this second coiled coil domain, suggesting that BicDR itself does not have an activated or inactivated state or that this is controlled through a different mechanism. A second dissimilarity between *BicD* and *BicDR* is the big difference between their expression levels. According to FlyBase (Jenkins et al., 2022), *BicDR* is mainly expressed in tracheae, gut, salivary glands and carcass tissue, whereas the expression in other tissues remains at low levels. Although there is some overlap with the expression of *BicD*, the expression of *BicDR* was described to remain low in the tissues where the consequences of *BicD* mutations have been described. Such tissues are the ovary, the young embryo and the nervous system (Aradska et al., 2015; Sanghavi et al., 2016; Vazquez-Pianzola et al., 2011). This means that we can assume that the expression of BicDR at low levels does not necessarily contribute to cargo transport the same way as BicD does. How could BicDR then support BicD? It does not appear to dimerize with a BicD subunit based on immunopurification or yeast two-hybrid results (Fig. S3, Tables S1, S2). *BicD* is more important in large cells where it transports cargo over very long distances. *BicDR* might be specialized for moving cargo for local transport over short distances (e.g. from the cell body into the bristle shaft). In cells where both are expressed, BicDR could then make the cargo more accessible for long-distance transport by BicD. This seems consistent with the function we found in the growing bristle shaft, where BicDR seems more involved in bringing Spn-F from the cell body to the shaft and BicD then acts to transport it toward the tip (Fig. 4). Although the proper tip localization of Spn-F depends on *BicD* and *BicDR*, we found that in the sensitized background (*BicD^PA66^*/−) both copies of *BicDR* are needed to move normal levels of Spn-F from the cell body into the bristle shaft (Fig. 5C). On the other hand, full *BicD* activity is not required for this step (Fig. 5C) but is required to obtain strong bristle tip localization of Spn-F (Fig. 5B). The hypothesis that BicDR contributes more to the short-distance transport to the base of the shaft and BicD more to the long-distance transport to the tip would also explain why *BicDR* bristles tend to be thinner in the proximal region close to the bristle base and *BicD* bristles thicker than the wild type. In the former situation, bristle construction factors would not make it into the bristle shaft, in the latter, they would be moved to the base of the bristle but fail to be transported away from the base.

*spn-F* is needed for the localization of Hook at the bristle tip (Bitan et al., 2010b) and *hook* is not only required for endocytic trafficking within the eye and the nervous system but also at the bristle tip. Given that there is evidence that endocytosis is responsible for the polarized transfer of lipids and membrane proteins, which again is necessary for the polarization of the bristle cell (Rodriguez-Boulan et al., 2005), our results point to an important contribution of BicD and BicDR to bristle development by localizing Spn-F to the tip. With Spn-F also being part of the IKKε–jvl complex, which regulates the shuttling movement of recycling endosomes and cytoskeletal organization (Otani et al., 2015), the lack of Spn-F in this complex would interfere with the shuttling regulation of motor proteins at the molecular signaling centers (Otani et al., 2015). This also prevents the transport of Rab-positive vesicles. Mutations in *Rab6* and *Rab11*, members of the Rab protein family that mediate intracellular vesicle trafficking, lead to impaired bristle growth (Purcell and Artavanis-Tsakonas, 1999; Khodosh et al., 2006; Zhang et al., 2007), and a *Rab6* bristle phenotype has been described that matches the bristle phenotype of *BicD^PA66^*/−; *BicDR^71^*/+ flies even at the SEM level (Fig. 2H; Purcell and Artavanis-Tsakonas, 1999). The accumulation of Rab6 signal at the bristle tip is in line with the description of the distal tip being the signaling center for bristle elongation and thereby the most dynamic part of the polarized cell (Otani et al., 2015). Reduced Rab6 levels at the distal tip in hemizygous *BicD* hypomorphic flies with either one or two functional copies of *BicDR* indicates that exocytosis and endocytosis at the bristle tip are impaired. Because Rab6 levels are strongly reduced in these mutants (Fig. 4C; Fig. S5), it is not clear whether the reduced Rab6 levels, reduced tip localization or both lead to the observed phenotype. However, because bristle growth takes place in different parts of the bristle (Fei et al., 2002), it seems that both defects could interfere with normal bristle formation.

Rab11 contributes to the construction of the bristle by inserting chitin synthase into the plasma membrane, thereby allowing bristle chitinization (Adler, 2020 preprint). With a complete lack of chitin synthase in *Rab11^−^* bristles, the bristles not only appear shorter but collapse completely. Even *BicD*; *BicDR* double mutants did not show significant length differences in bristle length during the pupal stage, suggesting that the limiting step in these animals is the construction of the final macrochaetae with their complete chitinization. Because the *Rab11* phenotype is quite different from the *BicD*; *BicDR* double mutant bristle phenotype, we did not focus on possible interactions with *Rab11*. However, the knowledge gained from this study might also be able to explain the *BicD*; *BicDR* double mutant bristle phenotype as being the result of a requirement for proper Rab11 localization in the bristle shaft and tip. Reduced transport of Rab11 (or a co-factor) toward the tip might cause a polar reduction of chitin incorporation towards the tip, causing an increased probability of breakage in the distal parts under reduced *BicD* and *BicDR* activity.

Actin modules in the bristle shaft are central to the construction of the bristle. The disorganized F-actin network seen in the mutants (Fig. 4B) can either be caused by insufficient build-up, maintenance or stability of F-actin, resulting in fragmented actin bundles or incorrect alignment of already formed bundles. These defects would be expected to prevent normal chitinization. Future research should address whether defects in Arp2/3 or Rab vesicle transport and localization cause the defective actin bundles and whether this affects chitinization and bristle shaft stability and causes shorter and thinner bristles.

Our results demonstrate how directed transport contributes to the organization of elongated and asymmetric cells. Microtubule transport, which localizes factors that organize cellular functions, connects directly and indirectly to vesicle trafficking and the stability of the actin cytoskeleton. We have shown that *BicD* and *BicDR* contribute together to this directed transport and the development of the long bristles in a partially redundant manner. Our results led to the hypothesis that BicD might be more specialized for long-haul transport and BicDR more for short-distance local transport. Future studies should test this hypothesis.

## MATERIALS AND METHODS

### Fly stocks and genetics

Flies were kept and bred on standard cornmeal agar containing yeast, sucrose, potassium sodium tartrate, methylparaben and propionic acid. For the crosses, multiple virgins (5 to 10) were added to several males (3 to 5) and incubated at 25°C. Fly strains used are listed in Table S3. *BicD^PA66^* (*BicD^A40V^*; Schüpbach and Wieschaus, 1991; Suter and Steward, 1991), *BicDR^(rev)^*, *BicDR^29^*, *BicDR^51^,* and *BicDR^71^*. Standard methods were used to generate *BicDR* excision stocks with the two *P* elements *P{SUPor-P} and P{RS5}* (Bellen et al., 2004; Ryder et al., 2004). The excisions were characterized molecularly by extracting DNA from heterozygous mutant males followed by PCR with primers framing the deleted regions of the *BicDR* gene. The screening by PCR revealed that two excision stocks are missing a fragment around the insertion site of the P-elements *P{SUPor-P}*: *BicDR^29^*, *BicDR^51^*, and one around the insertion site of *P{RS5}*: *BicDR^71^* (Fig. S1). These stocks were double-balanced and kept for further examinations. In the case of the mutant *BicDR^(rev)^*, the activated P-element *P{RS5}* reverted the genomic sequence of the *BicDR* gene to the wild-type sequence when it jumped out. The wild-type revertant *BicDR^(rev)^* was used as a control for the excision mutants.

*v; CyO/Sp* flies were kindly provided by Simon Bullock (Division of Cell Biology, Medical Research Council Laboratory of Molecular Biology, Cambridge, UK). Stocks from the Vienna Drosophila RNAi Center (VDRC) and the Bloomington Stock Center are listed in Table S3. For tissue-specific knockdown or gene expression, the *UAS-Gal4* system was used (Brand and Perrimon, 1993).

### CRISPR/Cas9 and generation of transgenic flies

All gRNAs were designed manually and verified on the web-based tool called *CRISPR* optimal target finder (https://flycrispr.org/target-finder/). The gRNAs for attaching a GFP-tag (5′-ATTATCGCTGAAATAAACTC-3′) and the gRNA for the deletion and substitution of K555 (5′-AGTCCATTCAGCAAAAGG-3′) were cloned into pCFD5 plasmids (Port and Bullock, 2016; kindly provided by Simon Bullock) following the ‘gRNA cloning protocol for cloning single gRNA plasmids’ protocol published previously (Port and Bullock, 2016). Transgenic flies were generated using the ΦC31-based integration system (Bischof et al., 2007) and crossed with *nos-Cas9*-expressing animals.

To add a GFP tag to the C-terminus of the BicDR protein, the appropriate *eGFP* DNA sequence (Tsien, 1998) with a linker and two 1200 bp long arms homologous to the *BicDR* gene and framing the stop codon were cloned into a pBluescript II SK (+) vector (Stratagene, now Agilent; La Jolla, CA, USA). The construct was injected into embryos with the genotype w, y, w^+^ *nos-Cas9*/Y; gRNA *v^+^*/+; BicDR*/BicDR*. To generate the *BicDR^K555A^::GFP* mutant, the sequence within the template vector was modified by site-directed mutagenesis before injection. All constructs were sequence verified. All primers used for DNA construction are listed in Table S4.

### Genetic interaction assay

Crosses were female *w*; *BicD^PA66^*/CyO; BicDR* / TM6B×*w*; *Df7068*/CyO; *Df4515/*TM3, Sb male, where BicDR* indicates one of the excisions, *BicDR^29^*, *BicDR^51^* and *BicDR^71^*, the deletion mutant *BicDR^8.1^*, the *BicDR^null^* allele *Df737* (*BicDR^Df^*) or the wild-type revertant *BicDR^(rev)^*.

For every cross, 30 virgin females were added to 15 males. Every 2 days, the flies were transferred to a new plastic bottle. The frequency of every genotype of the progeny was determined. The progeny was also sorted by sex and genotype and kept at 18°C for the following experiments. Females who were no longer not virgins anymore were dissected, and their ovaries were stained. The frequency of genotypes of eclosed flies from each cross was counted for 9 days each. All statistics and graphics were made using the GraphPad Prism 5 software.

### Analysis of bristle development

The following crosses were used to determine the severity of the *BicDR* alleles and their interaction with *BicD*:

female *w*; *BicD^PA66^*/CyO; BicDR*/TM6B×*Df7068*/SM6B male to give *w*; *BicD^PA66^*/*Df7068*; BicDR* /+.

female *w*; *BicD^PA66^*/CyO ftz lacZ×*w*; *Df7068*/CyO; *Df4515*, *w^+^/*TM3, Sb male to give *w*; *BicD^PA66^*/*Df7068*; *Df4515, w^+^*/+.

To investigate the bristle phenotype further, pupae with the following genotypes were dissected and stained following the protocol by Tilney et al. (1998). The outline of the bristle cell was estimated from the distribution of the actin bundles and the cell body from the position of the root (basis) of the actin bundles (i.e. the cell body of the bristle cell is expected to be directly beneath the first actin bundles).

female *w*; *BicD^PA66^*/ CyO, Act-GFP; BicDR*/TM6B, Tb×Df7068/CyO, Act-GFP male, to give *w*; *BicD^PA66^*/*Df7068*; BicDR*/+ and *w*; *BicD^PA66^*/ *Df7068*; TM6B, Tb/+.

### Immunostaining and microscopy

Dechorionated embryos or dissected tissue that was kept on ice for less than 30 min was fixed in 4% paraformaldehyde for 20 min and blocked with either 5% milk or bovine serum albumin (Fraction V) for 2 h at room temperature. Primary antibodies were incubated overnight, followed by washing steps and incubation with secondary antibodies for at least 2 h. Primary antibodies were diluted as follows: anti-GFP (rabbit, 1:200, 210-PS-1GFP, ImmunoKontact), anti-GFP (mouse, 1:200, MA5-15256, Thermo Fisher Scientific), anti-Ef1γ (rat, 1:1000; Serpinskaya et al., 2014), anti-Rab6 (rabbit and guinea pig, 1:200; Iwanami et al., 2016), anti-Spn-F (rabbit, 1:300, AB_10570329, DSHB) and anti-Asense (guinea pig, 1:100; Brand et al., 1993). Secondary antibodies were conjugated to Alexa Fluor 488 (anti-rabbit-IgG 1:800), Alexa Fluor 488 Plus (anti-rabbit-IgG, 1:200), Alexa Fluor 647 (anti-mouse-IgG, 1:200), Alexa Fluor 647 Plus (anti-rabbit-IgG, 1:200) and Cy3 (anti-mouse-IgG, 1:400). DNA staining was for 20 min with 2.5 mg/ml of Hoechst 33258 during the final wash step. The images were taken with a Leica TCS-SP8 confocal laser-scanning microscope and processed using FIJI software.

### Scanning electron microscopy of bristles

Flies were anesthetized with CO_2_, decapitated, mounted and coated with gold. Scanning was performed on a Zeiss Gemini 450 SEM with electron high tension set to 5 kV. Signals were detected with detectors for secondary electrons (and backscattered electrons). For measuring the length of the bristles, only apparently intact bristles that were mounted relatively horizontally were considered. In all cases, only the length of the longer pSC bristles was recorded because these were apparently the ones that were mounted more horizontally and the measurements on the pictures could be expected to be more accurate.

### Isolation of embryonic BicDR complexes for mass spectrometry

Embryos (12–16 h old) were collected and lysed in homogenization buffer (25 mM HEPES pH 7.4, 150 mM NaCl, 0.5 mM EDTA and 1 mM DTT and 1 tablet of proteinase inhibitor cocktail; Roche 11836170001). The aqueous phase of the lysate was collected after 1 h of centrifugation at 21,300 ***g*** at 4°C and centrifugation again for 25 min. Subsequently, one part of the aqueous phase was saved as input control, whereas the rest was incubated with Plus Sepharose G beads that were coated with anti-GFP antibody overnight at 4°C, following the protocol of Vazquez-Pianzola et al. (2017). 5–7 washing steps with wash buffer (25 mM HEPES pH 7.4, 150 mM NaCl, 0.5 mM EDTA, 1 mM DTT and a half tablet of proteinase inhibitor cocktail; Roche 11836170001) were performed before the beads were either sent for mass spectrometry or prepared with the appropriate amount of SDS for SDS-PAGE and western blot analysis. SDS/PAGE bands that were present in the IP from the BicDR::GFP fusion protein were cut out of the gel and sent directly for mass spectrometry at the Proteomics and Mass Spectrometry Core Facility of the University of Bern. As a control, the equivalent regions of the control lanes were also cut out and used for a mass spectrometric analysis.

### Yeast two-hybrid assay

The full-length cDNA of *BicDR* and *Ef1γ* as well as the C-terminal domain (CTD) of *BicDR* were cloned into pOAD and pOBD2 vectors so that they were in frame with the activator domain (AD) or the DNA binding domain (BD) (Cagney et al., 2000; Vazquez-Pianzola et al., 2022). In this way, BicDR-AD, BicDR-CTD-AD, Ef1γ-AD, as well as BicDR-BD, BicDR-CTD-BD, and Ef1γ-BD were created. The BicD-AD and Egl-AD, as well as BicD-BD and Egl-BD, have been described previously (Vazquez-Pianzola et al., 2022).

## Supplementary Material



10.1242/joces.261408_sup1 Supplementary information

Table S5. Differential expression analysis of proteins that were identified in either the tagged BicDR::GFP IP, BicDRK555A::GFP or in the wild-type negative control IP (MS analysis).

Table S6. Proteins identified in BicDR::GFP immunoprecipitations by MS analysis of SDS-PAGE bands.
