# Supplementary material for: Role of BicDR in bristle shaft construction and support of BicD functions
Source: J Cell Sci. 2024 Jan 31;137(2):jcs261408. doi: 10.1242/jcs.261408 (PMC10917063; doi:10.1242/jcs.261408)
Supplement: Supplementary information [file joces-137-261408-s1.pdf]

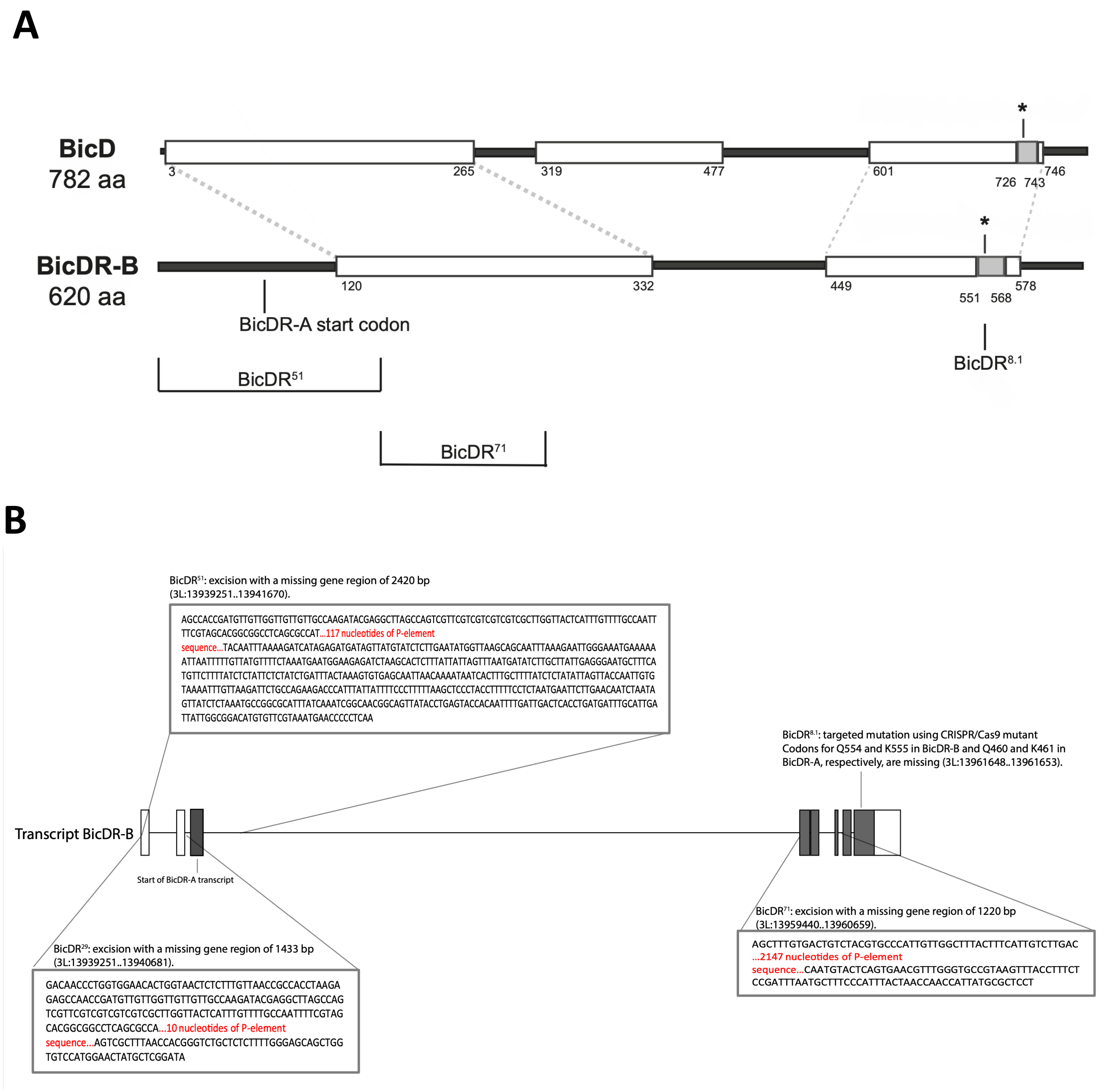

Fig. S1. Comparing BicD and BicDR structures and the *BicDR* gene A) Structure comparison of the *Drosophila* proteins BicD and BicDR. Shown in open boxes are the coiled coil domains, in filled boxes the sequence with the highest homology. The lysine with a key role in cargo attachment is indicated with a star. It is localized at position 730 in BicD, at position 555 in BicDR-B, and 461 in BicDR-A. The regions altered in the different alleles are indicated. The hemizygous deletion *BicDR*$^{8.1}$ removes precisely the two codons Q554 and K555 in *BicDR*. **B)** Structure of *Drosophila BicDR-A* and *–B* mRNAs and the excision mutants. The gray boxes frame the parts of the gene that have been removed by the imprecise P-element excisions. The sequences in the box show the position of the excision breakpoints. Sequences in red are P-element leftovers. The excision *BicDR*^{29} misses the 5' UTR region, without impairing the first protein-coding exon, while *BicDR*^{51} misses the first protein coding exon of *BicDR-A* and *–B* and the 5' UTR region as well. *BicDR*^{71} is the only excision that removes protein coding exons 2, 3, and 4 but leaves exons 5 and 6 intact. *BicDR*$^{8.1}$ is a CRISPR mutant with deleted Q554 and K555 codons.

Journal of Cell Science • Supplementary information

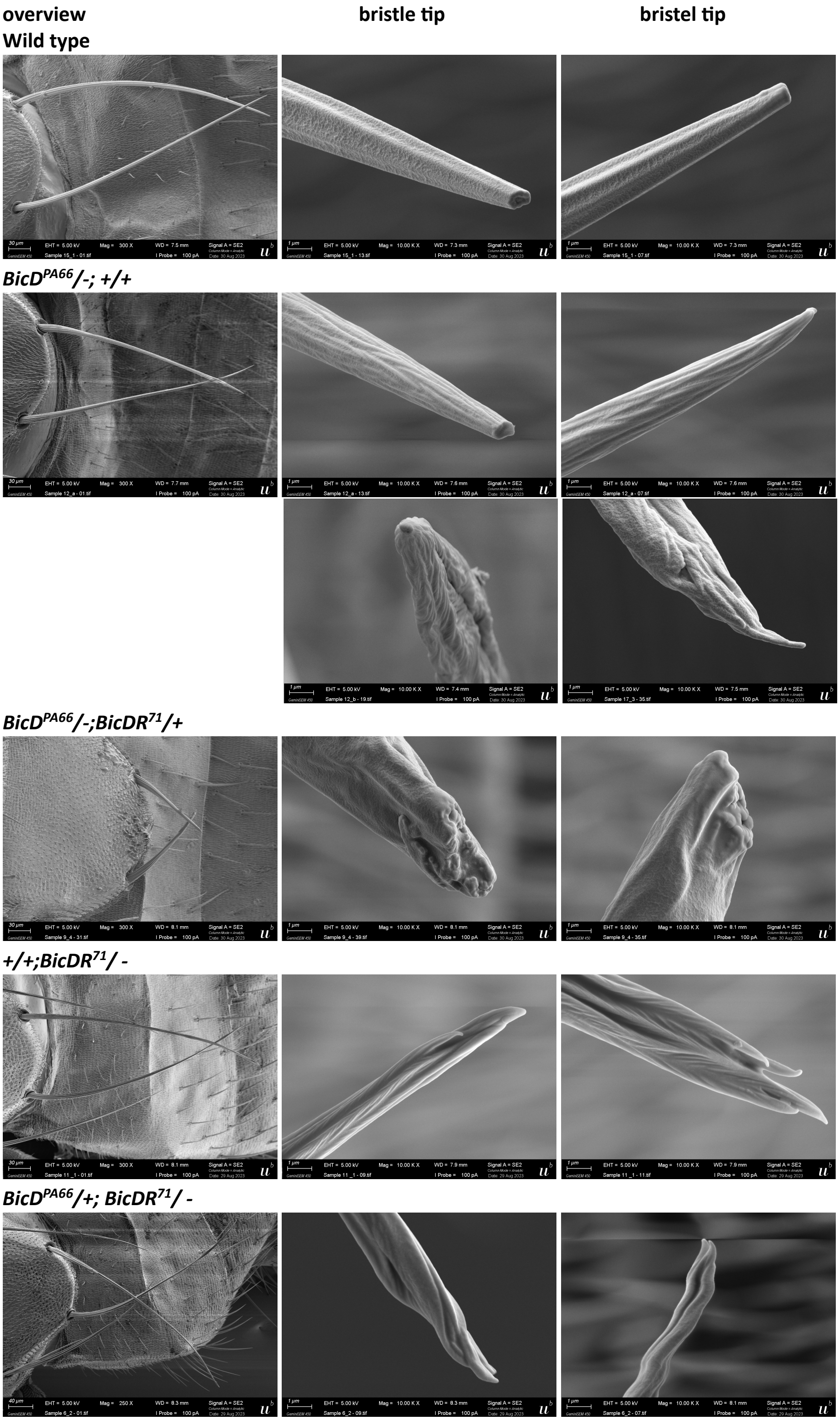

Fig. S2. Posterior scutellar bristles (pSC): overview (left) and corresponding bristle tips. The genotypes are indicated above the overview and the corresponding tips to the right. The 2nd chromosomes with the *BicD* gene and the 3rd with the *BicDR* gene are indicated. *BicD* alleles were *BicD*^{PA66} (PA66) and Df7068 (-). *BicDR* alleles were *BicDR*^{71} and Df4515 (-). "+" indicates a wild-type allele of *BicD* or *BicDR* either on an uncharacterized chromosome or a balancer chromosome. Additional examples from these and other *BicDR* allelles have been deposited at the Dryad Digital Repository (Suter et al., 2024; https://doi.org/10.5061/dryad.dfn2z358t).

Journal of Cell Science • Supplementary information

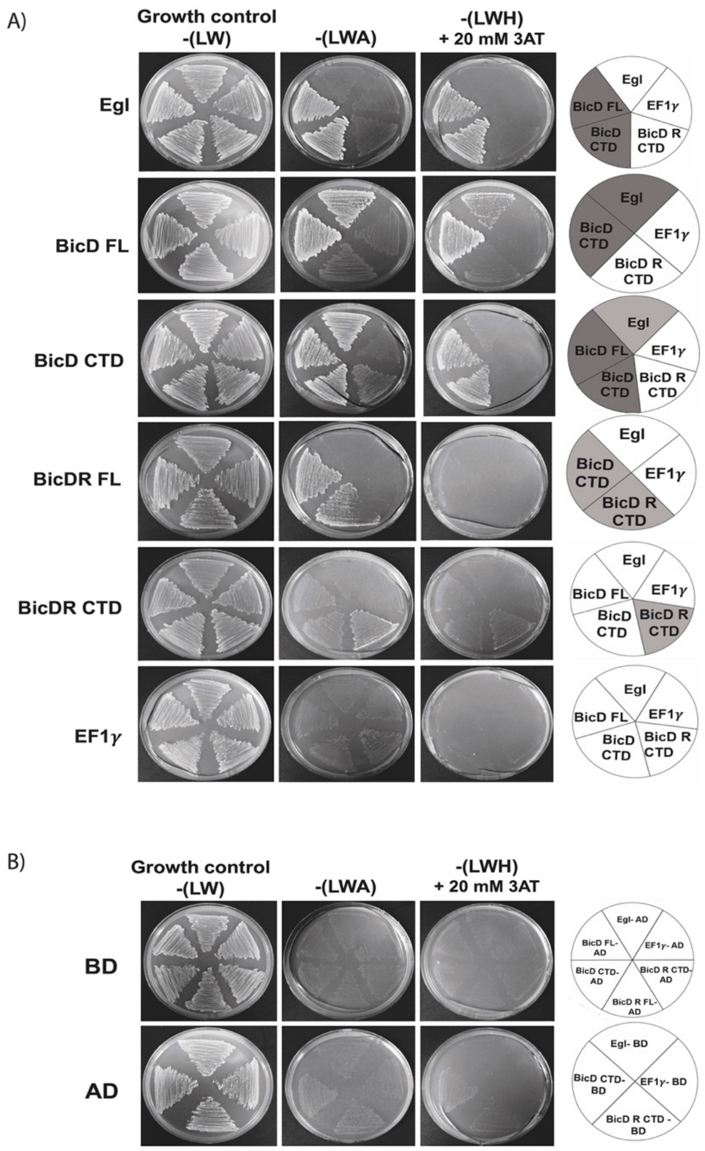

Fig. S3. *Drosophila* BicDR does not interact with EF1γ, Egl, or BicD. (A) The activation domain is indicated on the left and the binding domains are on the right side. The latter is drawn where these activation domains were plated out. (-LW): grown on medium selecting for the two plasmids, (-LWH): selective plates on which cells with the activator domain and the DNA binding domain clones can grow if they weakly interact. (-LWH) with 20 mM 3-aminotriazole [3-AT]: selective plates on which cells with the activator domain and the DNA binding domain clones can grow if they strongly interact. FL: full length, CTD: C-terminal domain, BD: binding domain, AD: activation domain. BicD and Egl were used as positive controls since their interaction had already been demonstrated[79]. Here, the full-length protein of BicDR, fused to the activation domain, binds to the C-terminal domain of BicDR as well as to the C-terminal domain of BicD. However, this result is not confirmed under stringent conditions, since neither the full-length BicD protein nor the C-terminal domain of BicD seems to bind to BicDR in immunoprecipitations nor according to the yeast 2-hybrid assay. Thus, these results seem to indicate that the direct interaction between BicDR FL (AD) and BicD (BD) resulted from the nonspecific entanglement of the coiled coil domains[80]. **(B)** Negative controls.

Journal of Cell Science • Supplementary information

Wild-type examples

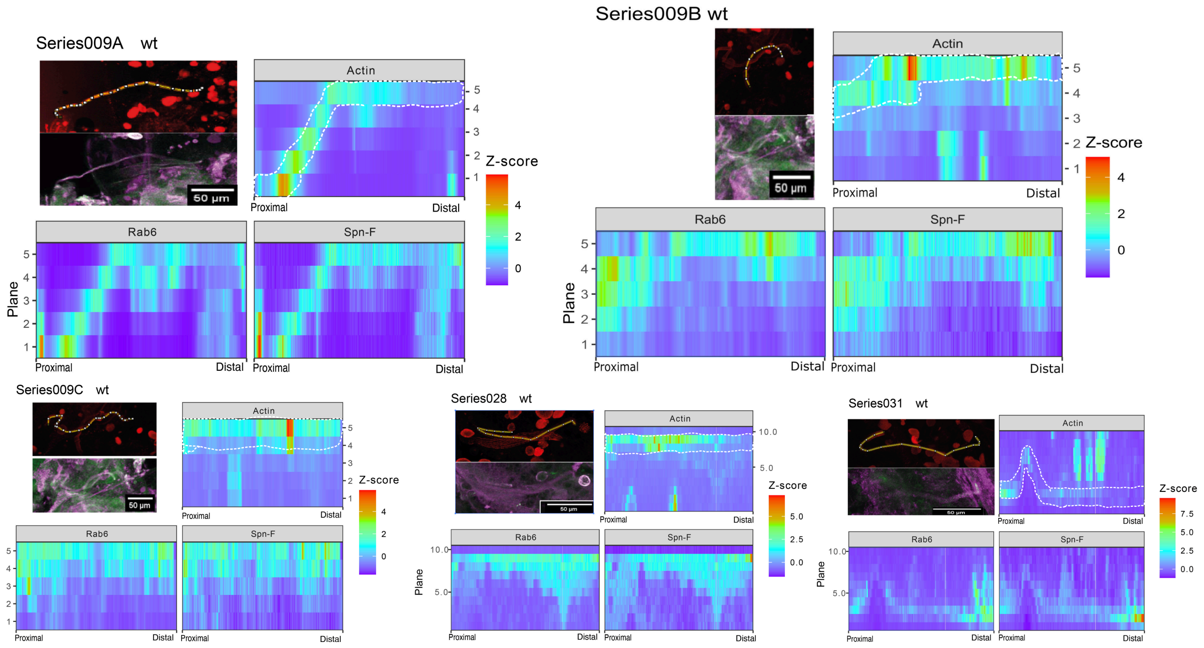

BicD^{PA66}/- examples

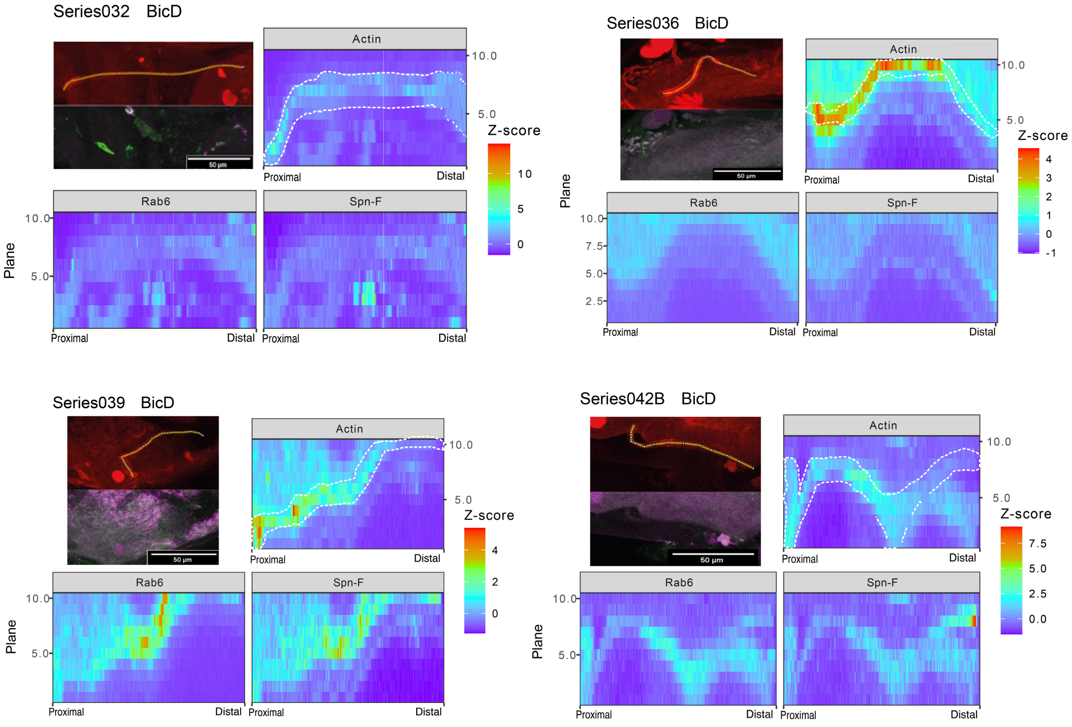

Journal of Cell Science • Supplementary information

BicD^{PA66}/-; BicDR^{71}/+ examples

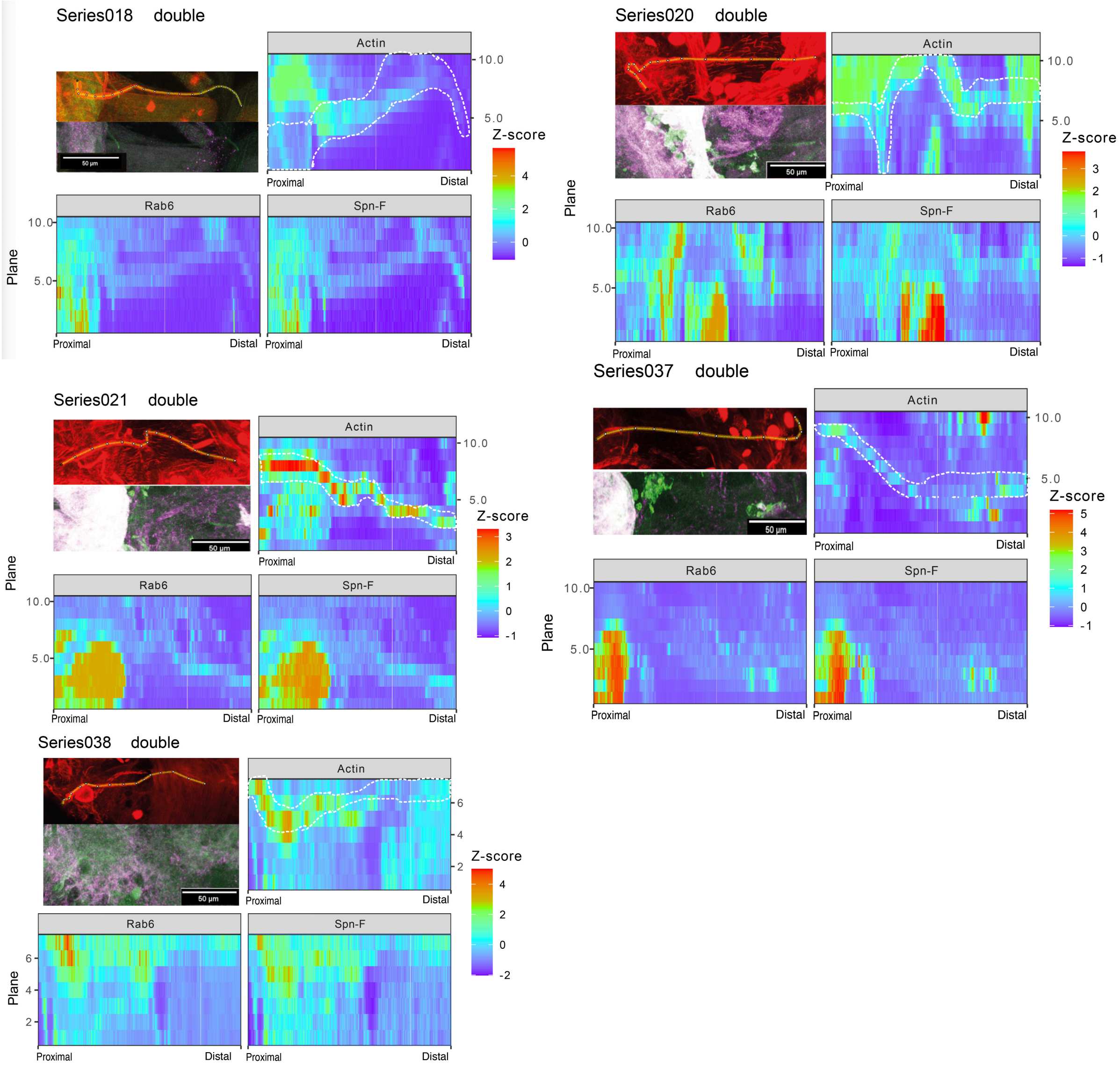

Fig. S4 (Display of signal intensity is normalized for each channel). This shows the distribution of the indicated signals; but levels cannot be compared between channels. The top left picture shows a maximal projection of the region with the pupal posterior Scutellar Bristle (pSC). On top, the F-actin signal and a drawing of the line along which the staining intensities of the three channels were measured. Below, the staining for Spn-F in green and Rab6 in red. Pictures are oriented such that the proximal end of the bristle shaft is to the left side and the distal one to the right. The other three panels show the intensity of the signal in the different z-planes along the drawn line. The approximate position of the bristle shaft was estimated from the F-actin (Actin) and the Spn-F signal and is shown with a dashed line in the F-actin panel. The methods are described in the main part of the paper.

Wild-type examples

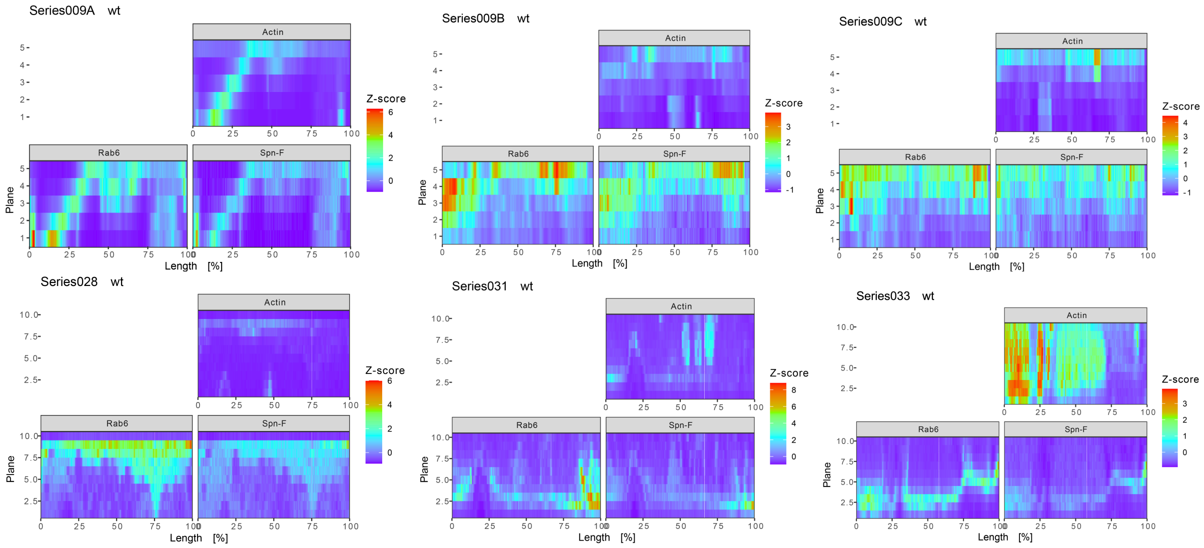

BicD^{PA66}/- examples

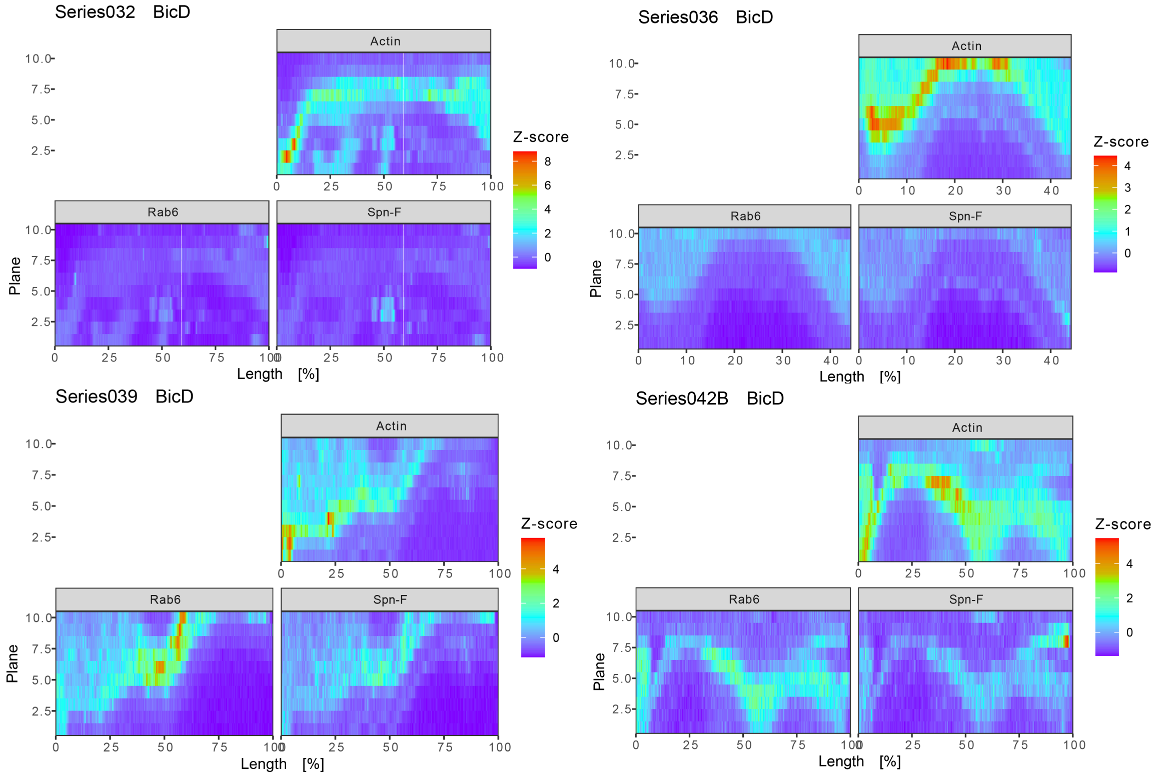

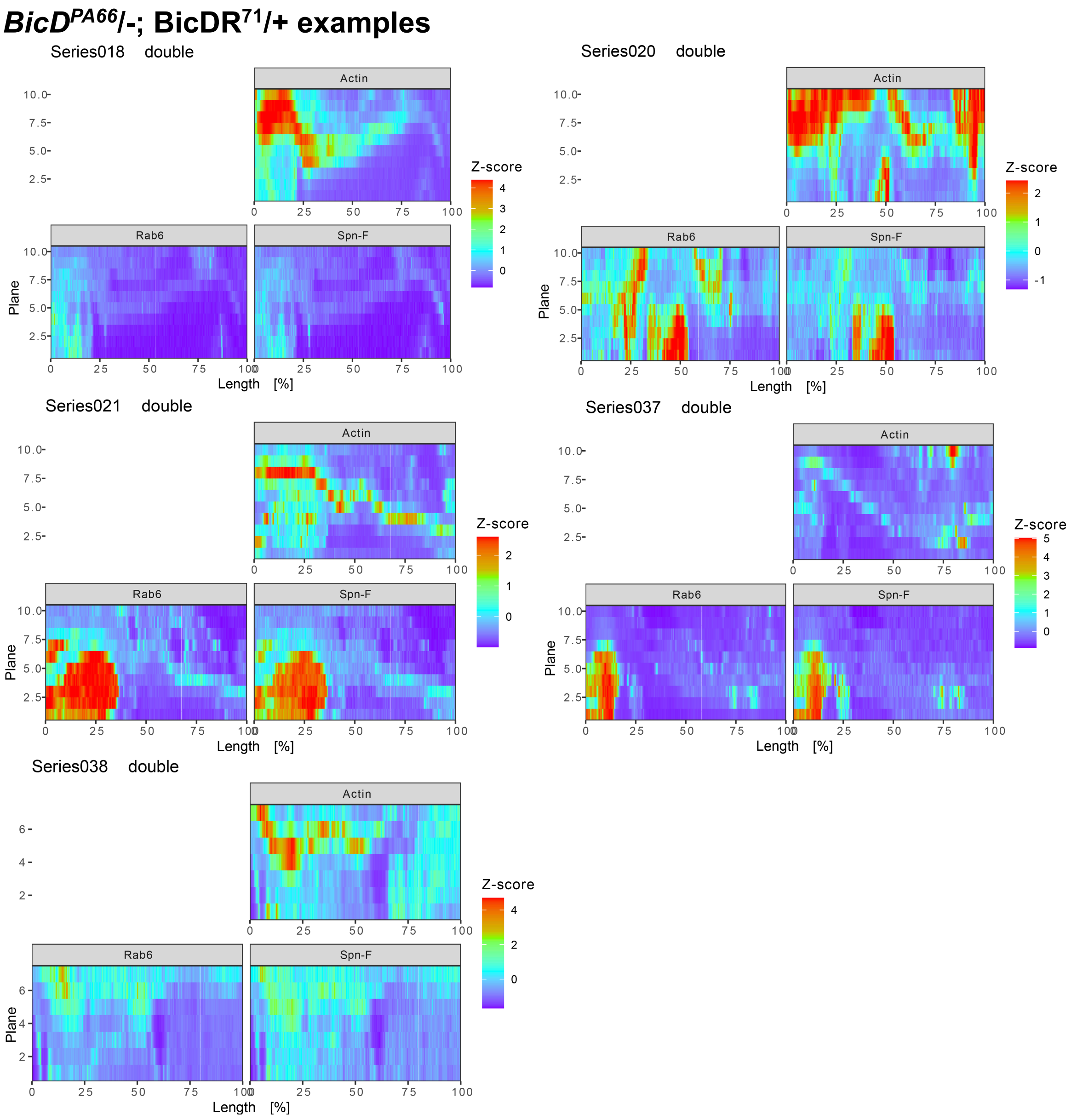

Fig. S5. Same data as in Figure S4 but with signal intensities normalized over the 3 channels. Taking the background signals into consideration, this allows to compare Rab6 and Spn-F expression levels along the bristle shaft across genotypes relative to F-actin. The maximal projection image and the approximate outline of the pSC through the z-stack planes are depicted in the corresponding top panels of Figure S4. Graphs are oriented such that the proximal end of the bristle shaft is to the left and the distal one to the right. The three panels show the intensity of the signal in the different z-planes measured along the pupal bristle (see Figure S4). The methods are described in the main part of the paper.

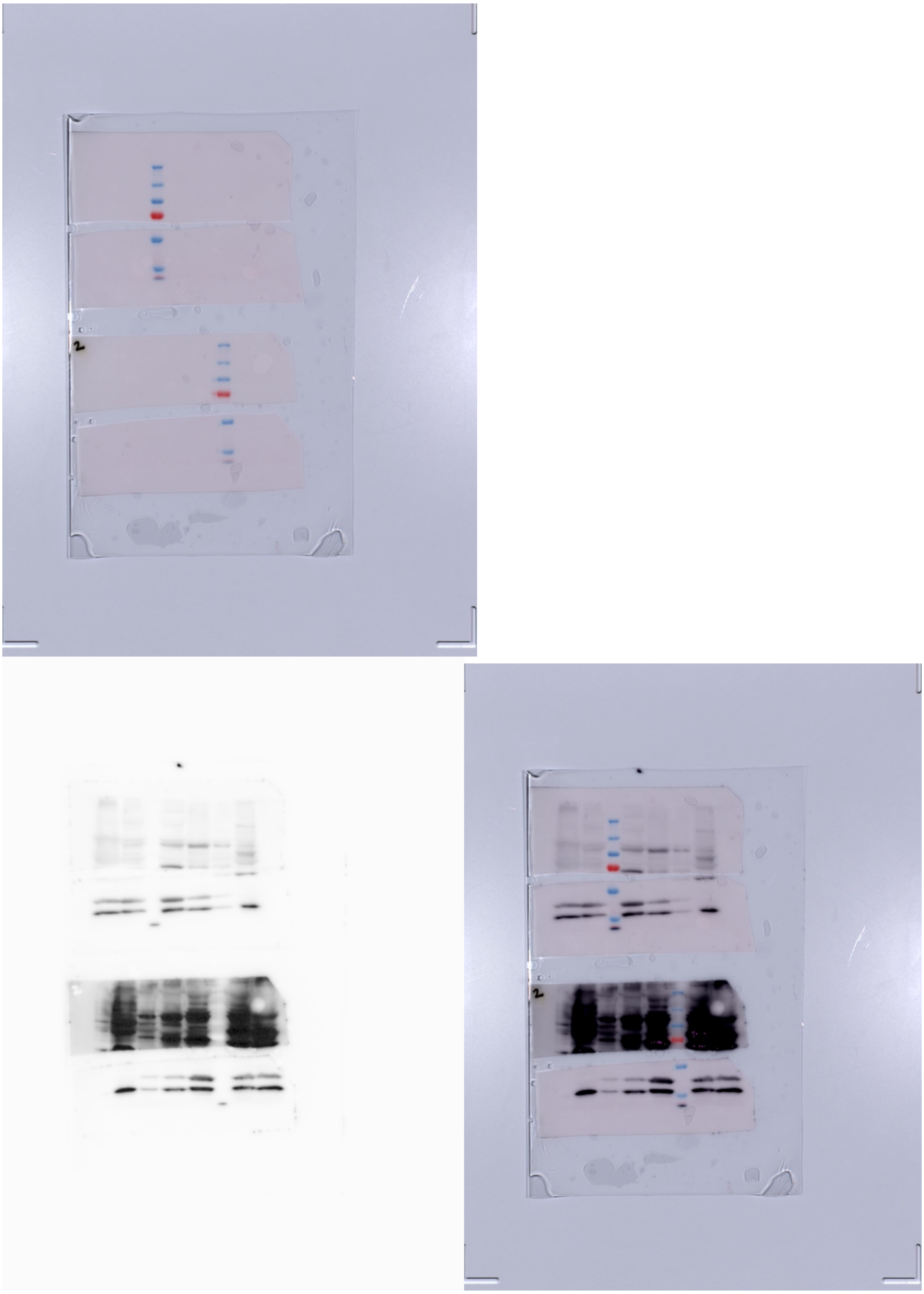

Fig. S6. Blot transparency

Journal of Cell Science • Supplementary information

Table S1. Proteins that were significantly enriched in the tagged BicDR::GFP IPs in comparison to the wild-type negative control IPs. Genes encoding these proteins are listed according to their level of significance of enrichment over the control. The abbreviations used are “control” for the *white* control, “BicDR” (BicDR::GFP), and “BicDR$^{K>A}$” (BicDR^{K555A}::GFP). Hits enriched in tagged wild-type BicDR compared to BicDR$^{K>A}$ are listed as potential cargo, whereas peptides enriched in tagged wild- type BicDR and BicDR^{K555A} IPs are listed as potential non-cargo interactors. GN is the abbreviation for gene name. The iBAQ equals the sum of all peptide intensities divided by the number of observable peptides of a protein[1]. $\log_2$FC is the logarithm of the mean ratio between the two groups and the adjusted p-value (adj. pVal) highlights the factor level comparisons within a family that are significantly different[2,3]. -1 and -2: indicate different biological replicates.

GN	Sequence coverage [%]	iBAQ	log2FC BicDR - control	log2FC BicDR$^{K>A}$ - control	log2FC BicDR$^{K>A}$ - BicDR	Adj. pVal BicDR - control	Adj. pVal BicDR$^{K>A}$ - control	Adj. pVal BicDR$^{K>A}$ - BicDR	Pot. Cargo	Pot. Non-cargo interactor
**BicDR**	84.7	1.70E+09	11.401	11.470	0.069	0.000	0.000	0.998	-	-
**Phk-3**	21.5	8.34E+06	3.745	3.478	-0.267	0.000	0.002	0.998		Yes
**Dmel\CG10211**	2.9	3.08E+05	3.546	4.039	0.493	0.000	0.000	0.998		Yes
**Hsp67Bc**	23.6	2.29E+06	3.513	4.133	0.620	0.002	0.002	0.998		Yes
**Rac1**	12.5	3.32E+06	3.039	1.662	-1.377	0.001	0.300	0.998	Yes	
**BicDR::GFP**	59.9	1.85E+07	2.804	3.021	0.217	0.002	0.024	0.998	-	-
**l(2)k01209**	4.6	1.11E+06	2.670	0.707	-1.963	0.015	0.774	0.998	Yes	
**Kri**	15.3	1.25E+07	2.242	1.486	-0.756	0.017	0.103	0.998	Yes	
**RpS5a**	49.6	2.85E+08	2.181	1.771	-0.410	0.024	0.099	0.998	Yes	
**tou**	1.8	2.80E+05	2.015	-0.341	-2.357	0.004	0.880	0.998	Yes	
**Mtl**	10.8	1.03E+07	1.796	1.192	-0.604	0.047	0.121	0.998	Yes	
**mey**	8.0	4.32E+06	1.741	0.498	-1.243	0.005	0.648	0.998	Yes	
**Srp54k**	35.0	2.57E+07	1.706	1.563	-0.143	0.004	0.024	0.998		Yes
**RpS4**	63.2	2.97E+08	1.689	1.373	-0.317	0.004	0.076	0.998	Yes	
**RpL12**	52.1	5.18E+08	1.680	1.538	-0.142	0.047	0.103	0.998	Yes	
**RpS5b**	43.5	1.11E+09	1.529	1.406	-0.123	0.013	0.036	0.998		Yes
**RpS19a**	96.2	3.66E+09	1.366	1.288	-0.078	0.047	0.100	0.998	Yes	
**RpL27A**	53.7	1.30E+08	1.315	1.234	-0.081	0.021	0.100	0.998	Yes	
**RpS17**	84.0	1.53E+09	1.290	1.210	-0.079	0.016	0.103	0.998	Yes	
**RpL35**	35.0	2.15E+08	1.289	0.872	-0.417	0.047	0.196	0.998	Yes	
**Gp93**	18.2	1.07E+07	1.273	0.688	-0.585	0.021	0.316	0.998	Yes	
**RpL13**	62.8	5.56E+08	1.143	0.826	-0.317	0.047	0.205	0.998	Yes	
**RpL31**	79.8	3.79E+08	1.104	0.915	-0.189	0.047	0.121	0.998	Yes	
**RpS18**	73.7	1.42E+09	1.095	0.983	-0.112	0.047	0.112	0.998	Yes	

Table S2. Out of the 118 proteins identified in the SDS-PAGE bands of BicDR::GFP immunoprecipitations, 21 are known to result in bristle phenotypes if perturbed. Abbreviations for BicDR::GFP: BicDR and $BicDR^{K555A}$::GFP: $BicDR^{K>A}$. The Sum PSM describes the summarized number of peptide spectrum matches of the sample with the indicated genotype. Primary data is from Table S6.

Description	Percent Coverage	Protein length	Sum PSM BicDR_1	Sum PSM BicDR_2	Sum PSM $BicDR^{K>A}$_1	Sum PSM $BicDR^{K>A}$_2	Reference for bristle phenotype
**B52**	7.6	355	1	3	0	0	4
**RpS3A**	12.6	246	0	3	0	0	5
**Cpb**	10.9	276	0	4	0	0	6
**RpS17**	19.1	131	0	2	0	0	7
**Arp3**	31.6	418	10	3	0	0	8
**RpS3A**	40.7	268	9	15	0	0	9
**Orc6**	23.0	257	2	5	0	0	10
**RE01362p (Fragment)**	30.0	299	0	14	0	0	11
**Orc4**	16.3	459	6	1	0	0	12
**Vha26**	52.2	226	22	18	0	0	13
**Rumi**	5.7	316	1	0	0	0	14
**Par-6**	29.6	351	7	1	0	0	15
**Ef1γ**	52.0	431	80	43	6	0	16
**NAT1**	5.6	1488	0	1	3	4	17
**Arm**	2.0	843	5	4	1	0	18
**Parvin**	12.0	367	3	1	2	0	19
**CtBP**	10.3	476	0	2	0	1	20
**Hrb27C**	27.3	421	17	14	1	0	17
**Hsp83**	2.9	717	4	4	2	0	21
**Sam-S**	24.0	408	0	7	1	0	22
**Arp2**	22.8	399	0	9	2	2	23

References for Tables S1-2

1. Fabre, B. *et al.* Comparison of label-free quantification methods for the determination of protein complexes subunits stoichiometry. *EuPA Open Proteomics* **4**, 82–86 (2014).
2. Wright, S. P. Adjusted P-Values for Simultaneous Inference. *Biometrics* **48**, 1005 (1992).
3. Wang, M. *et al.* Amino acid metabolism, lipid metabolism, and oxidative stress are associated with post-stroke depression: A metabonomics study. *BMC Neurol.* **20**, 1–10 (2020).

Journal of Cell Science • Supplementary information

4. Kraus, M. E. & Lis, J. T. The concentration of B52, an essential splicing factor and regulator of splice site choice in vitro, is critical for Drosophila development. *Mol. Cell. Biol.* **14**, 5360–5370 (1994).

5. Sæbøe-Larssen, S., Lyamouri, M., Merriam, J., Oksvold, M. P. & Lambertsson, A. Ribosomal Protein Insufficiency and the Minute Syndrome in Drosophila: A Dose-Response Relationship. *Genetics* **148**, 1215–1224 (1998).

6. Hopmann, R. & Miller, K. G. A balance of capping protein and profilin functions is required to regulate actin polymerization in Drosophila bristle. *Mol. Biol. Cell* **14**, 118–128 (2003).

7. Hart, K., Klein, T. & Wilcox, M. A Minute encoding a ribosomal protein enhances wing morphogenesis mutants. *Mech. Dev.* **43**, 101–110 (1993).

8. Rajan, A., Tien, A. C., Haueter, C. M., Schulze, K. L. & Bellen, H. J. The Arp2/3 complex and WASp are required for apical trafficking of Delta into microvilli during cell fate specification of sensory organ precursors. *Nat. Cell Biol.* **11**, 815–824 (2009).

9. Casad, M. E. *et al.* Cardiomyopathy is associated with ribosomal protein gene haplo-insufficiency in Drosophila melanogaster. *Genetics* **189**, 861–870 (2011).

10. Balasov, M., Akhmetova, K. & Chesnokov, I. Humanized drosophila model of the Meier-Gorlin syndrome reveals conserved and divergent features of the Orc6 protein. *Genetics* **216**, 995–1007 (2020).

11. Zielke, N., Vähärautio, A., Liu, J., Kivioja, T. & Taipale, J. Upregulation of ribosome biogenesis via canonical E-boxes is required for Myc-driven proliferation. *Dev. Cell* **57**, 1024-1036.e5 (2022).

12. McDaniel, S. L. *et al.* Tissue-Specific DNA replication defects in drosophila melanogaster caused by a meier-gorlin syndrome mutation in orc4. *Genetics* **214**, 355–367 (2020).

13. Le Bras, S., Rondanino, C., Kriegel-Taki, G. raldine, Dussert, A. & Borgne, R. Le. Genetic identification of intracellular trafficking regulators involved in Notch-dependent binary cell fate acquisition following asymmetric cell division. *J. Cell Sci.* **125**, 4886–4901 (2012).

14. Acar, M. *et al.* Rumi Is a CAP10 Domain Glycosyltransferase that Modifies Notch and Is Required for Notch Signaling. *Cell* **132**, 247–258 (2008).

15. Besson, C. *et al.* Planar cell polarity breaks the symmetry of PAR protein distribution prior to mitosis in Drosophila sensory organ precursor cells. *Curr. Biol.* **25**, 1104–1110 (2015).

16. Fan, Y. *et al.* Drosophila Translational Elongation Factor-1γ Is Modified in Response to DOA Kinase Activity and Is Essential for Cellular Viability. *Genetics* **184**, 141–154 (2010).

17. Mummery-Widmer, J. L. *et al.* Genome-wide analysis of Notch signalling in Drosophila by transgenic RNAi. *Nature* **458**, 987–992 (2009).

18. Lin, X. & Perrimon, N. Dally cooperates with Drosophila Frizzled 2 to transduce Wingless signalling. *Nature* **400**, 281–284 (1999).

19. Chountala, M., Vakaloglou, K. M. & Zervas, C. G. Parvin Overexpression Uncovers Tissue-Specific Genetic Pathways and Disrupts F-Actin to Induce Apoptosis in the Developing Epithelia in Drosophila. *PLoS One* **7**, (2012).

20. Stern, M. D. *et al.* CtBP is required for proper development of peripheral nervous system in Drosophila. *Mech. Dev.* **126**, 68–79 (2009).

Journal of Cell Science • Supplementary information

21. Milton, C. C., Batterham, P., McKenzie, J. A. & Hoffmann, A. A. Effect of E(sev) and Su(Raf) Hsp83 mutants and trans-heterozygotes on bristle trait means and variation in Drosophila melanogaster. *Genetics* **171**, 119–130 (2005).

22. Larsson, J. & Rasmuson-Lestander, Å. Somatic and germline clone analysis in mutants of the S-adenosylmethionine synthetase encoding gene in Drosophila melanogaster. *FEBS Lett.* **427**, 119–123 (1998).

23. Koch, N. *et al.* Abp1 utilizes the Arp2/3 complex activator Scar/WAVE in bristle development. *J. Cell Sci.* **125**, 3578–3589 (2012).

Table S3. Publicly available fly stocks used in this study.

BL stands for "Bloomington Stock Center" and VDRC for "Vienna RNAi Center".

Name	ID
37870	VDRC# 37870
32137R 2 III	VDRC# 32137R 2 III
Df(3L)ED4515	BL# 9071
Df(3L)BSC737	BL# 26835
Df(2L)Exel7068	BL# 7838
nos-Cas9	BL# 54591
nos-phiC31 int	BL# 25709
eEF1gamma[A42]	BL# 40962
eEF1gamma[A70]	BL# 40963
eEF1gamma[A28]	BL# 40964
eEF1gamma[A15]	BL# 40965
Df(3R)Exel6212	BL# 7690

Table S4. Primers used in this study.

The primers listed in this table were used for the generation of *BicDR::GFP* and *BicDR^{K555A}::GFP* expressing flies and for the yeast 2-hybrid experiment. as described in the methods section.

Name	Sequence	Application
BicDR homologous Arm 1 fwd	5'- atcgtcgGCGGCCGCCTCCTAAATGGATTTCTAACTAACC-3'	Generation of *BicDR::GFP* expressing flies
BicDR homologous Arm1 rvs	5'- ACTTCCGGATCCTGCTCGCTGAAATAAACTC-3'	Generation of *BicDR::GFP* expressing flies
GFP primer 1 fwd	5'- CGAGCAGGATCCGGAAGTGGATCTATGGTGAGCAAGGGCGAG-3'	Generation of *BicDR::GFP* expressing flies
GFP primer 1 rvs	5'- CTCCAAGAATTCTTACTTGTACAGCTCGTCCA-3'	Generation of *BicDR::GFP* expressing flies
BicDR homologous Arm 2 fwd	5'- AAGTAAGAATTCTTGGAGTAACCTAACGACCCGCTGG-3'	Generation of *BicDR::GFP* expressing flies
BicDR homologous Arm 2 rvs	5'- cgacgatGGGCCCGCACGTGGACAATAATGA-3'	Generation of *BicDR::GFP* expressing flies
Mutagenesis Primer for BicDRK555A fwd	5'- GCAAGCGGTCGAGCTGTCAC-3'	Generation of *BicDR^{K555A}::GFP* expressing flies
Mutagenesis Primer for BicDRK555A rvs	5'- GTGACAGCTCGACCGCTTGC-3'	Generation of *BicDR^{K555A}::GFP* expressing flies
Screening fwd	5'- CCACATGAAGCAGCACGAC-3'	Generation of *BicDR::GFP* and *BicDR^{K555A}::GFP* expressing flies
Screening rvs	5'- CTACGAACAGAAGAAGGGCA-3'	Generation of *BicDR::GFP* and *BicDR^{K555A}::GFP* expressing flies
BicDR-full -Sense	5'- accaccatggcaattcccgggATGCATAAACCAAAGCTAGCGAA -3'	Yeast 2-Hybrid experiment
BicDR-full -Anti	5'- gcaggtcgacggatccccgggTTATCGCTGAAATAAACTCCAAAGC -3'	Yeast 2-Hybrid experiment
BicDR-CTD sense	5'- accaccatggcaattcccgggAGTTTGCAATTCGAAATGGAATG -3'	Yeast 2-Hybrid experiment
BicDR-CTD rvs	5'- gcaggtcgacggatccccgggTTATCGCTGAAATAAACTCCAAAGC -3'	Yeast 2-Hybrid experiment
eIF1gamma sense	5'- accaccatggcaattcccgggATGGTGAAAGGAACTCTGTACACTTACC -3'	Yeast 2-Hybrid experiment
eIF1gamma Anti	5'- gcaggtcgacggatccccgggTTACTTGAAGATCTTGCCCTGGT -3'	Yeast 2-Hybrid experiment

Table S5. Differential expression analysis of proteins that were identified in either the tagged BicDR::GFP IP, BicDRK555A::GFP or in the wild-type negative control IP (MS analysis).

Available for download at
https://journals.biologists.com/jcs/article-lookup/doi/10.1242/jcs.261408#supplementary-data

Table S6. Proteins identified in BicDR::GFP immunoprecipitations by MS analysis of SDS-PAGE bands.

Available for download at
https://journals.biologists.com/jcs/article-lookup/doi/10.1242/jcs.261408#supplementary-data
