# Supplementary material for: Role of BicDR in bristle shaft construction and support of BicD functions
Source: J Cell Sci. 2024 Jan 31;137(2):jcs261408. doi: 10.1242/jcs.261408 (PMC10917063; doi:10.1242/jcs.261408)
Supplement: Supplementary file 1 [file joces-137-261408_review_history.pdf]

Journal of Cell Science

Role of *BicDR* in bristle shaft construction and support of *BicD* functions

Aleksandra Jejina, Yeniffer Ayala, Dirk Beuchle, Thomas Höhener, Ruth E. Dörig, Paula Vazquez-Pianzola, Greco Hernández and Beat Suter

DOI: 10.1242/jcs.261408

Editor: David Stephens

Review timeline

Original submission:	16 June 2023
Editorial decision:	19 July 2023
First revision received:	2 November 2023
Editorial decision:	29 November 2023
Second revision received:	14 December 2023
Accepted:	15 December 2023

Original submission

First decision letter

MS ID#: JOCES/2023/261408

MS TITLE: Role of BicDR in bristle shaft construction, tracheal development, and support of BicD functions

AUTHORS: Aleksandra Jejina, Yeniffer Ayala, Greco Hernández, and Beat Suter

ARTICLE TYPE: Research Article

We have now reached a decision on the above manuscript.

To see the reviewers' reports and a copy of this decision letter, please go to: https://submit-jcs.biologists.org and click on the 'Manuscripts with Decisions' queue in the Author Area. (Corresponding author only has access to reviews.)

As you will see, the reviewers raise a number of substantial criticisms that prevent me from accepting the paper at this stage. They suggest, however, that a revised version might prove acceptable, if you can address their concerns. If you think that you can deal satisfactorily with the criticisms on revision, I would be pleased to see a revised manuscript. We would then return it to the reviewers.

Both reviewers highlight the need to provide further data to support the conclusions drawn but are enthusiastic about the work and I am therefore hopeful that you would be able to address their concerns.

Please ensure that you clearly highlight all changes made in the revised manuscript. Please avoid using 'Tracked changes' in Word files as these are lost in PDF conversion.

I should be grateful if you would also provide a point-by-point response detailing how you have dealt with the points raised by the reviewers in the 'Response to Reviewers' box. Please attend to

© 2023. Published by The Company of Biologists under the terms of the Creative Commons Attribution License (https://creativecommons.org/licenses/by/4.0/).

all of the reviewers' comments. If you do not agree with any of their criticisms or suggestions please explain clearly why this is so.

Reviewer 1

Advance summary and potential significance to field

Bicaudal D (BicD) is a critical component of the RNA transport of anterior determinants in Drosophila oogenesis. The research on this protein established the importance of adaptor molecules of dynein motor protein in RNA transport. BicD-like proteins were first discovered as proteins with domain organization somewhat similar to BicD and the role of RNA transport in mammalian cells. BicDR was found as a Drosophila counterpart of BicD-like. This paper investigated the genetic requirement of BicDR. They first reported that apparent null mutants of BicDR were viable and fertile with no clear defects, suggesting BicDR is dispensable for Drosophila development and survival. Then the authors asked BicD and BicDR act together by creating double mutation stocks (heterozygote of BicD over BicDR null condition). In this manuscript, phenotypes of adult bristles (aSC and pSC macrochaetae) were described. In addition, the phenotype of embryonic trachea and expression of BicDR, revealed by the genomic knock-in of GFP, were reported.

Comments for the author

Main Comments:
1) Bristle Phenotype. The authors demonstrated that a subset of adult macrochaetae displayed slightly shortened lengths and, occasionally, loss of pigmentation phenotypes (Fig. 1, 2). Given the significant drop in the viability of double mutants, the adult phenotypes described here must have been observed in a limited number of escapers. This limited sample size may have introduced bias into the analysis. One potential solution to this issue is the cell-specific knockdown of the two genes using the UAS-RNAi technique.

The authors then studied the macrochaetae at the pupal stage (40-44h APF). They showed that F-actin bundles appeared disorganized (Fig. 6B), and that bristle tip localization of IKK epsilon kinase and Rab6 was impaired (Fig. 6C, Fig. 7). As IKK epsilon had previously been shown to be transported by the dynein motor and regulates dynein cargo unloading, and its mutations significantly affect bristle morphology, it was hypothesized that IKK epsilon could be one of the downstream targets of BicD and BicDR. However, the morphological description of BicD BicDR mutant bristles was insufficient to allow for comparison with the previously reported IKK epsilon mutant bristle phenotypes. A detailed scanning electron microscopic analysis would be beneficial to determine if the bristle phenotypes of BicD BicDR mutants are similar to those of IKK epsilon mutants. Analyses of the pupal stage bristles were performed on pupae at 40-44h APF. At this stage, macrochaetae elongation is complete, and IKK epsilon localization is decreasing, making it too late to monitor IKK epsilon function effectively. An analysis of the 32-36h APF bristles would be more appropriate for monitoring IKK epsilon activity.

2) Interaction with Ef1b gamma. Among the candidate proteins associated with BicDR in fly tissues, the authors investigated Ef1b gamma, which functions in protein translation (Fig. 8). They found that heterozygosity of Ef1b gamma enhanced the bristle phenotype of BicD BicDR mutants. However, it remains unclear whether this interaction reflects the proposed role of BicDR on IKK epsilon or an entirely different pathway.

3) Tracheal Phenotype The authors examined tracheal morphology in stage 16 embryos and discovered that BicDR mutants showed occasional breakage of the dorsal trunk. This phenotype was further enhanced by removing one copy of BicD. However, their expression analysis with BicDR-GFP did not reveal any expression in the embryonic trachea (Fig. 4), making it challenging to understand BicDR's function in the tracheal system.

4) Expression of BicDR. The authors created a genomic knock-in of GFP to the C-terminus of BicDR to monitor its expression (Fig. 4). The reporter revealed clear expression in the embryonic salivary gland, particularly accumulating in the apical domain (Fig. 4B). The role of BicDR in the salivary gland was not reported in this work. BicDR-GFP was also detected in segmentally repeated clusters. The authors argued that this expression overlaps with that of neural precursors (Fig. 4C). However,

© 2023. Published by The Company of Biologists under the terms of the Creative Commons Attribution License (https://creativecommons.org/licenses/by/4.0/).

the poor image quality of Fig. 4C precludes a firm conclusion. This issue is also seen in Fig. 5, where they suggested BicDR-GFP is expressed in the macrochaetae precursors in third-instar larvae. To support the analysis of the bristle phenotype, expression of BicDR-GFP should be monitored in the bristle cells of 33-36h APF.

Overall, the authors have made extensive and commendable efforts to characterize the function of BicDR in several aspects of development, including bristle development, embryonic trachea, and the salivary gland. However, the current conclusions are not robust enough to satisfy the readers of the Journal of Cell Science (JCS).

Other Comments:
The lettering in some figures, such as Fig. 8, is too small to read comfortably.

In Fig. 2, the total number of flies counted should be included for clarity.

The graphs in Fig. 2B and C are rather complex. They could be simplified without losing any critical information.

Lines 243-246: The authors dismiss the model of BicD/BicDR heterodimerization based on the absence of interaction in immunoprecipitation (IP) and yeast two-hybrid experiments. However, drawing such a conclusion from negative experiments seems premature.

In Fig. 3 and 4, the images with the DAPI channel do not appear to provide additional information. These could be removed or modified for clarity.

Reviewer 2

Advance summary and potential significance to field

This paper reports on the phenotypes and phenotypic interactions between BicD-Related (BicDR) and Bicaudal-D (BicD), a highly conserved component of the transport machinery that moves cargo (vesicles) toward the minus ends of microtubules. As part of a complex with Dynein and Dynactin, BicD links cargo to the motor. BicDR varies slightly in structure from BicD in that it has only two coiled-coil domains, as opposed to BicD, which has a third central coiled-coil domain that regulates transport function.

In this study, a series of loss-of-function alleles of BicDR were created using two different P-element insertions in the gene, including what appears to be a null allele. Complete loss of BicDR does not affect viability or fertility, and the associated phenotypes include subtle defects in bristle length and pigmentation and small gaps in the embryonic trachea. BicDR mutations show genetic interactions with loss of function mutations in BicD in increasing the severity of the bristle phenotypes as well as the severity of defects in the embryonic trachea, and decreasing organismal viability. The authors show that BicDR form homo- but not hetero-dimers with BicD. Based on the organization of actin filaments in the pupal bristles and localization of proteins that are normally found in the distal tips of the pupal bristles, the authors conclude that both proteins are required to transport cargo along microtubules but that BicDR transports cargo locally and BicD over longer distances. Finally, the authors identify many potential interacting (cargo?) proteins using a couple of approaches. They follow up on one of these proteins EF1γ, a translation elongation factor, and demonstrate genetic interactions with BicDR mutations.

These findings suggest that BicDR function could overlap/complement that of BicD in microtubule-dependent trafficking (pending some additional experiments).

Comments for the author

Overall concerns/issues:

© 2023. Published by The Company of Biologists under the terms of the Creative Commons Attribution License (https://creativecommons.org/licenses/by/4.0/).

1. From the tracheal staining, one cannot learn much about the phenotype and how it might be linked to cargo transport. The authors should stain the mutants (and corresponding controls) with a nuclear marker and another tracheal cell marker so that one can know if tracheal cells are present and simply failing to form connections or whether the cells are not there. Alternatively the tracheal cells could be failing to secrete the lumenal material that is being assayed (something that would fit with the trafficking function being proposed for BicDR).
2. The other concern with the trachea experiments is that the BicDR71 allele is homozygous in these experiments when it should have been used in trans to either a deficiency or other unrelated strong allele of BicDR. As the authors know, homozygosity for an entire chromosome makes it unclear if it is the lesion they know about that matters or whether there is some other unknown mutation on the chromosome that is responsible (indeed, the authors themselves discuss this possibility in the context of bristle duplication defects).
3. They are also missing the control of BicDPA66/+ without any lesion in BicDR.
4. The other concern regarding the tracheal defects is that the authors have not shown any expression of BicDR in the embryonic trachea, even when they stain for GFP with their endogenously tagged version of the gene. On the other hand, BicD mRNA is provided maternally and does appear to be expressed in the trachea based on BDGP data, which is one reason they need to show that reduced BicD alone is not the basis for the more severe tracheal defects. modEncode data showing high expression in third instar larval trachea does not mean the gene is expressed in the embryonic trachea.
5. Another issue that needs resolving is whether the BicDR-GFP protein functions like the WT protein – does homozygosity for this allele or this allele in trans to a deficiency have any of the bristle phenotypes reported for the loss of function alleles?
6. In Figure 6/7, there are a couple of issues. One is that there is no quantification for Rab6 localization in WT versus different mutant combinations.
Also, it is completely unclear what the authors are using as a marker for the
“cell bodies” of these bristle cells. It doesn’t seem the cell bodies are marked with either Actin or Spn-F. This is an important issue since they use this data to conclude that BicDR is involved in local transport and BicD acts over a long distance.

Other suggestions:
1. It would be very helpful to have the figure showing the molecular lesions with the different BicDR alleles in the paper rather than in a supplement. Along with the information provided on the regions deleted in the DNA, showing what is, in theory, deleted in the corresponding BicDR protein would also be helpful to the reader. The authors might also consider a cartoon of BicD protein structure as well.
2. It would be good to know the molecular lesion in the BicDPA66 allele.
Is it a problem with expression of the BicD gene/protein, or are there domains missing?
3. Figure 2B/C is a bit confusing. Does BicDPA66/-;BicDRrev/+ have short bristles or not? It’s very difficult to know from either the images or the histograms as currently presented. I think it would be easier to understand the phenotypic frequency if the histogram in B were removed. Also, they only need to show one half of the histogram currently in C.
4. I’m not so sure about the relevance of the BicDR-GFP expression in the larval imaginal wing disc since the functions of the protein are probably needed much later during pupal stages. Does BicDR-GFP colocalize with Ase in the bristle precursors in pupal discs?
5. The explanation in Lines 222 to 224 is quite confusing.
6. Where does BicDR8.1 fall in their allelic series of severity? Would be good to include and to use for the tracheal experiments.

First revision

Author response to reviewers' comments

Reviewer 1 Advance Summary and Potential Significance to Field:
Bicaudal D (BicD) is a critical component of the RNA transport of anterior determinants in Drosophila oogenesis. The research on this protein established the importance of adaptor molecules of dynein motor protein in RNA transport. BicD-like proteins were first discovered as proteins with

© 2023. Published by The Company of Biologists under the terms of the Creative Commons Attribution License (https://creativecommons.org/licenses/by/4.0/).

domain organization somewhat similar to BicD and the role of RNA transport in mammalian cells. BicDR was found as a Drosophila counterpart of BicD-like. This paper investigated the genetic requirement of BicDR. They first reported that apparent null mutants of BicDR were viable and fertile with no clear defects, suggesting BicDR is dispensable for Drosophila development and survival. Then the authors asked BicD and BicDR act together by creating double mutation stocks (heterozygote of BicD over BicDR null condition). In this manuscript, phenotypes of adult bristles (aSC and pSC macrochaetae) were described. In addition, the phenotype of embryonic trachea and expression of BicDR, revealed by the genomic knock-in of GFP, were reported.

Reviewer 1 Comments for the Author:
Main Comments:
1) Bristle Phenotype. The authors demonstrated that a subset of adult macrochaetae displayed slightly shortened lengths and, occasionally, loss of pigmentation phenotypes (Fig. 1, 2). Given the significant drop in the viability of double mutants, the adult phenotypes described here must have been observed in a limited number of escapers. This limited sample size may have introduced bias into the analysis. One potential solution to this issue is the cell-specific knockdown of the two genes using the UAS-RNAi technique.

➔ Only the double mutants with a hypomorphic *BicD* allele over deficiency and *BicDR* over deficiency are lethal (and the few escapers show a strong bristle phenotype). In former Figure 2B we wanted to point out this detail. But this obviously caused a misunderstanding not the least because our former Figure 2 is too complicated and former Figure 2B was also not labeled properly (we are grateful that this mistake did not slip through!). It does not show “Frequency of animals with bristle phenotype”, but actually “Frequency of surviving animals” (from the cross - which is the same as 2A) and that all survivors showed a bristle phenotype. Reviewer 2 also pointed out that we should improve this figure. Accordingly, we have removed former Figure 2B. We already had mentioned in the figure legends of former Figure 2A and in the main text that all escapers had short bristles. We, therefore, do not lose any information by deleting it.
For the analysis of the strength of the different *BicDR* alleles in bristle development, we had used the sensitized *BicD* background and the different *BicDR** alleles as heterozygotes (former Figure 2C) because this genotype is viable, we had good numbers and could compare the frequency of short bristles to assess the severity of the phenotype ($BicDR^{71}$ and $BicDR^{8.1}$ behaved as null mutants and $BicDR^{29}$ and $BicDR^{51}$ as hypomorphs). For these different alleles, we also added the numbers analyzed for each genotype. Furthermore, we also observed a bristle phenotype in *BicDR* mutants (former Figure 1C, D) and RNAi knock downs of *BicDR* in the former Figure 1 E-F. The new EM analysis (present Fig. 2) provides a good number of additional insights.

The authors then studied the macrochaetae at the pupal stage (40-44h APF). They showed that F-actin bundles appeared disorganized (Fig. 6B), and that bristle tip localization of IKK epsilon kinase and Rab6 was impaired (Fig. 6C, Fig. 7). As IKK epsilon had previously been shown to be transported by the dynein motor and regulates dynein cargo unloading, and its mutations significantly affect bristle morphology, it was hypothesized that IKK epsilon could be one of the downstream targets of BicD and BicDR. However, the morphological description of BicD BicDR mutant bristles was insufficient to allow for comparison with the previously reported IKK epsilon mutant bristle phenotypes. A detailed scanning electron microscopic analysis would be beneficial to determine if the bristle phenotypes of BicD BicDR mutants are similar to those of IKK epsilon mutants. Analyses of the pupal stage bristles were performed on pupae at 40-44h APF. At this stage, macrochaetae elongation is complete, and IKK epsilon localization is decreasing, making it too late to monitor IKK epsilon function effectively. An analysis of the 32-36h APF bristles would be more appropriate for monitoring IKK epsilon activity.

➔ We are a bit confused with this issue. We studied the interaction with *spn-F*/SpnF, not with *IKK epsilon* (the two are known to interact) and have therefore used the conditions from the *spn-F* publication from the Abdu laboratory which used the 40-44h APF (Bitan et al. describe in their paper that the localization of Spn-F is still enriched at the bristle tips in wild-type 40-44h APF bristles[1]). In this way, we could compare our data with the published one on Spn-F. (The lack of Spn-F localization also leads to the lack of IKK epsilon localization (Otani et al., 2015), but we have never examined the *BicD* and *BicDR* mutant bristles for IKK epsilon. Because we also wanted to study when the mutant bristles start to differ in length, we have chosen the later window of 40-44h

© 2023. Published by The Company of Biologists under the terms of the Creative Commons Attribution License (https://creativecommons.org/licenses/by/4.0/).

APF seemed the best choice. At 40hs, wild-type bristles have grown three quarters of their final length and will stop elongating 48h APF[2].

As suggested by this reviewer, we have complemented and extended our data with the suggested scanning electron microscopic analyses and compared the bristle phenotype to the one of other known bristle genes. The similarity with a *rab6* mutant phenotype (present Fig. 2) at the EM level was stunning.

2) Interaction with Ef1b gamma. Among the candidate proteins associated with BicDR in fly tissues, the authors investigated Ef1b gamma, which functions in protein translation (Fig. 8). They found that heterozygosity of Ef1b gamma enhanced the bristle phenotype of BicD BicDR mutants. However, it remains unclear whether this interaction reflects the proposed role of BicDR on IKK epsilon or an entirely different pathway.

➔ This is true and it was not our intention to suggest such a connection. We will make sure that the text is clear about this. To be complete, we wish to mention that EF1 gamma also regulates organelle transport along microtubules (Genetics 184(1): 141--154; J. Cell Sci. 127(1): 33-39). We think that this function might be more important for its interaction with BicDR/BicD, but intentionally left open the question of whether BicDR and EF1 gamma function in the same pathway. In the manuscript we had written “Presumably because of the high signal levels in all tissues, one would need to use a more complex approach to test this.” We now changed this to “Presumably because of the high signal levels in all tissues, one would need to use a more complex approach to test whether EF1γ can be linked more closely to *BicD* and *BicDR* activity.” This should make it clear that we did not imply to have shown this.

3) Tracheal Phenotype The authors examined tracheal morphology in stage 16 embryos and discovered that BicDR mutants showed occasional breakage of the dorsal trunk. This phenotype was further enhanced by removing one copy of BicD. However, their expression analysis with BicDR-GFP did not reveal any expression in the embryonic trachea (Fig. 4), making it challenging to understand BicDR's function in the tracheal system.

➔ We agree with this assessment. We have agonized about including it in the manuscript, reducing it to a supplement or dropping it altogether. Given that the tracheal parts are incomplete and that controls are missing, we have decided that we will drop this part altogether and expand a bit more on the bristle phenotype. This point was also raised by reviewer 2. Deleting the tracheal part also included changing the title.

4) Expression of BicDR. The authors created a genomic knock-in of GFP to the C-terminus of BicDR to monitor its expression (Fig. 4). The reporter revealed clear expression in the embryonic salivary gland, particularly accumulating in the apical domain (Fig. 4B). The role of BicDR in the salivary gland was not reported in this work. BicDR-GFP was also detected in segmentally repeated clusters. The authors argued that this expression overlaps with that of neural precursors (Fig. 4C). However, the poor image quality of Fig. 4C precludes a firm conclusion. This issue is also seen in Fig. 5, where they suggested BicDR-GFP is expressed in the macrochaetae precursors in third-instar larvae. To support the analysis of the bristle phenotype, expression of BicDR-GFP should be monitored in the bristle cells of 33-36h APF.

➔ We have looked for defects in the salivary glands of *BicDR* mutant L3 larvae but failed to identify any. We, therefore decided to focus on the tracheae and the bristles, instead. We agree it is odd not to bring this up and mention now in the revised version that we failed to observe a salivary phenotype.
➔ In embryos, the peak staining intensity for BicDR::GFP (cytoplasmic) and Ase (nucelar; neuronal precursor marker) does not overlap. It strongest staining cells appear more likely to be neighboring cells. We have stated this now more clearly to avoid misinterpretations. As in the wing disk cells (former Figure 5), BicDR::GFP still appears to be expressed in the Ase positive cells, but it does not peak there, which would have been nice to see). Considering that expression in a specific cell does not imply function in this cell, we focused on studying the effect on described players of bristle construction, Spn-F and Rab6, instead. Furthermore, we plan to go with the suggestion of reviewer 2 and drop the staining of the larval disc (former Figure 5). Together with this, we also deleted the description of the similarities between the larval and adult mechanosensory organs.

© 2023. Published by The Company of Biologists under the terms of the Creative Commons Attribution License (https://creativecommons.org/licenses/by/4.0/).

Other Comments:
The lettering in some figures, such as Fig. 8, is too small to read comfortably.

- ✓ We enlarged the text in most figures.

  In Fig. 2, the total number of flies counted should be included for clarity.

- ✓ We added the information.

  The graphs in Fig. 2B and C are rather complex. They could be simplified without losing any critical information.

- ✓ Done

  Lines 243-246: The authors dismiss the model of BicD/BicDR heterodimerization based on the absence of interaction in immunoprecipitation (IP) and yeast two-hybrid experiments. However, drawing such a conclusion from negative experiments seems premature.

➔ With our previous phrasing, we had not tried to pretend that we ruled out this possibility. Nevertheless, we have further weakened the conclusion by stating now: “Therefore, our results did not provide evidence that BicDR supports BicD function by forming dimers.”
In Fig. 3 and 4, the images with the DAPI channel do not appear to provide additional information. These could be removed or modified for clarity.

- ✓ This is another very good point. We removed the DNA staining for most panels. For Figure 3, we dropped the three panels and for Figure 4, we dropped this channel in some of them and added a panel with DNA staining for another.
  This should be better now.

*****
Reviewer 2 Advance Summary and Potential Significance to Field:
This paper reports on the phenotypes and phenotypic interactions between BicD-Related (BicDR) and Bicaudal-D (BicD), a highly conserved component of the transport machinery that moves cargo (vesicles) toward the minus ends of microtubules. As part of a complex with Dynein and Dynactin, BicD links cargo to the motor. BicDR varies slightly in structure from BicD in that it has only two coiled-coil domains, as opposed to BicD, which has a third central coiled-coil domain that regulates transport function.

In this study, a series of loss-of-function alleles of BicDR were created using two different P-element insertions in the gene, including what appears to be a null allele. Complete loss of BicDR does not affect viability or fertility, and the associated phenotypes include subtle defects in bristle length and pigmentation and small gaps in the embryonic trachea. BicDR mutations show genetic interactions with loss of function mutations in BicD in increasing the severity of the bristle phenotypes as well as the severity of defects in the embryonic trachea, and decreasing organismal viability. The authors show that BicDR form homo- but not hetero-dimers with BicD. Based on the organization of actin filaments in the pupal bristles and localization of proteins that are normally found in the distal tips of the pupal bristles, the authors conclude that both proteins are required to transport cargo along microtubules but that BicDR transports cargo locally and BicD over longer distances. Finally, the authors identify many potential interacting (cargo?) proteins using a couple of approaches. They follow up on one of these proteins EF1γ, a translation elongation factor, and demonstrate genetic interactions with BicDR mutations.

These findings suggest that BicDR function could overlap/complement that of BicD in microtubule-dependent trafficking (pending some additional experiments).

Reviewer 2 Comments for the Author:

© 2023. Published by The Company of Biologists under the terms of the Creative Commons Attribution License (https://creativecommons.org/licenses/by/4.0/).

Overall concerns/issues:

1.From the tracheal staining, one cannot learn much about the phenotype and how it might be linked to cargo transport. The authors should stain the mutants (and corresponding controls) with a nuclear marker and another tracheal cell marker so that one can know if tracheal cells are present and simply failing to form connections or whether the cells are not there. Alternatively, the tracheal cells could be failing to secrete the lumenal material that is being assayed (something that would fit with the trafficking function being proposed for BicDR).

2.The other concern with the trachea experiments is that the BicDR71 allele is homozygous in these experiments when it should have been used in trans to either a deficiency or other unrelated strong allele of BicDR. As the authors know, homozygosity for an entire chromosome makes it unclear if it is the lesion they know about that matters or whether there is some other unknown mutation on the chromosome that is responsible (indeed, the authors themselves discuss this possibility in the context of bristle duplication defects).

3.They are also missing the control of BicDPA66/+ without any lesion in BicDR.

4.The other concern regarding the tracheal defects is that the authors have not shown any expression of BicDR in the embryonic trachea, even when they stain for GFP with their endogenously tagged version of the gene. On the other hand, BicD mRNA is provided maternally and does appear to be expressed in the trachea based on BDGP data, which is one reason they need to show that reduced BicD alone is not the basis for the more severe tracheal defects. modEncode data showing high expression in third instar larval trachea does not mean the gene is expressed in the embryonic trachea.

➔ This reviewer convincingly argues that we should delete Figure 3. We had agonized about including it in the manuscript, reducing it to a supplement or dropping it altogether. Given that the tracheal parts are incomplete and that not all controls have been made, we have decided to drop this part altogether. This also takes care of an issue reviewer 1 made.

5.Another issue that needs resolving is whether the BicDR-GFP protein functions like the WT protein - does homozygosity for this allele or this allele in trans to a deficiency have any of the bristle phenotypes reported for the loss of function alleles?

➔ Homozygosity for the BicDR-GFP allele shows only wild-type bristles. (Numerous flies of this genotype were analyzed and all were normal). Over deficiency, 5 were analyzed and they were normal, too). In the revised manuscript we now mention that this alteration of *BicDR* does not affect the construction of the bristle.

6.In Figure 6/7, there are a couple of issues. One is that there is no quantification for Rab6 localization in WT versus different mutant combinations. Also, it is completely unclear what the authors are using as a marker for the “cell bodies” of these bristle cells. It doesn’t seem the cell bodies are marked with either Actin or Spn-F. This is an important issue since they use this data to conclude that BicDR is involved in local transport and BicD acts over a long distance.

➔ We added more quantitative analyses of Rab6, Spn-F, and F-actin distribution in the bristle by showing the signal profiles through the different z-planes for the different genotypes. In this way, we were able to analyze the levels of RAb6 and Spn-F expression relative to F-actin (it is strongly reduced if *BicD* and *BicDR* activity is reduced) and their distribution in the bristle shaft and tip (present Figs 4,5,S4).

We did not use a specific marker that would outline the precise cell body but used the roots/basis of the actin cables to pinpoint the cell body. We now wrote in the Methods section the following text. “The outline of the bristle cell was estimated from the distribution of the actin cables and the cell body from the position of the root (basis) of the actin cables (i.e., the cell body of the bristle cell is expected to be directly beneath the first F-actin bundles).” Additionally, we made the maximum projection pictures simpler and by displaying the actin channel in an independent panel together with the line along which the intensities were measure. In the adjacent panel, we now show the Rab6 and the Spn-F stainings for the same bristle.

Other suggestions:

© 2023. Published by The Company of Biologists under the terms of the Creative Commons Attribution License (https://creativecommons.org/licenses/by/4.0/).

1.It would be very helpful to have the figure showing the molecular lesions with the different BicDR alleles in the paper rather than in a supplement. Along with the information provided on the regions deleted in the DNA, showing what is, in theory, deleted in the corresponding BicDR protein would also be helpful to the reader. The authors might also consider a cartoon of BicD protein structure as well.

➔ In our mind, this should not be in the Figure because we have no idea what the chances are that a protein coding RNA is made and stable. We therefore left this part in the Supplementary Figure.

2.It would be good to know the molecular lesion in the BicDPA66 allele.
Is it a problem with expression of the BicD gene/protein, or are there domains missing?
➔ We have added this information with the references. *BicD*^{PA66}=*BicD*^{A40V} (in the Ser-Ala rich sequence SSASAQSA-40). It is still expressed.

3.Figure 2B/C is a bit confusing. Does BicDPA66/-;BicDRrev/+ have short bristles or not?
➔ Please note that in the former Figure 2B, the genotype was not *BicD*^{PA66}/-;*BicDR*^{rev}/+ but *BicD*^{PA66}/-;*BicDR*^{rev}/-. In the former Figure 2C, *BicD*^{PA66}/-;*BicDR*^{rev}/+ flies had normal bristles.
It's very difficult to know from either the images or the histograms as currently presented. I think it would be easier to understand the phenotypic frequency if the histogram in B were removed. Also, they only need to show one half of the histogram currently in C.
➔ We are very glad, that this reviewer put the finger on this.
Figure 2B caused a misunderstanding and was not labeled properly. We now removed former Figure 2B. We already had mentioned in the main text and in the figure legends of former Figure 2A that all escapers had short bristles. We, therefore, do not lose any information by deleting former Figure 2B. Also as suggested, in former Figure 2C we have deleted the half of the histogram that only shows the difference to 100%.

4.I'm not so sure about the relevance of the BicDR-GFP expression in the larval imaginal wing disc since the functions of the protein are probably needed much later during pupal stages. Does BicDR-GFP colocalize with Ase in the bristle precursors in pupal discs?
➔ We agree that this figure does not provide sufficient important information. We, therefore, removed this figure. The pupal Ase staining turned out more difficult and did not provide clear results.

5.The explanation in Lines 222 to 224 is quite confusing.
➔ By deleting former Figure 2B, we got rid of this issue.

6.Where does BicDR8.1 fall in their allelic series of severity? Would be good to include and to use for the tracheal experiments.
➔ *BicDR8.1* (is *BicDR*$^{\Delta GK555}$); the deletion of the cargo attachment Lys (plus the residue before the Lys) and it behaved as severe as the *BicDR*^{71} allele that we consider to be a null allele. We have now added this information to the description of the allelic series "*BicDR8.1* (=*BicDR*$^{\Delta GK555}$) also appeared to be a null allele like *BicDR*^{71}, but was not tested as much as the latter". This is because it was not made when we performed the initial characterizations of the mutants (e.g., the ones we used to analyze the effect on the tracheal system).

Second decision letter

MS ID#: JOCES/2023/261408

MS TITLE: Role of BicDR in bristle shaft construction and support of BicD functions

AUTHORS: Aleksandra Jejina, Yeniffer Ayala, Dirk Beuchle, Thomas C. Hoehener, Ruth E. Doerig, Paula Vazquez-Pianzola, Greco Hernandez, and Beat Suter
ARTICLE TYPE: Research Article

We have now reached a decision on the above manuscript.

© 2023. Published by The Company of Biologists under the terms of the Creative Commons Attribution License (https://creativecommons.org/licenses/by/4.0/).

To see the reviewers' reports and a copy of this decision letter, please go to: https://submit-jcs.biologists.org and click on the 'Manuscripts with Decisions' queue in the Author Area. (Corresponding author only has access to reviews.)

As you will see, the reviewers gave mixed reports but on balance I consider that a revised version would be acceptable for publication. The reviews do raise some additional points that will require amendments to your manuscript. I hope that you will be able to carry these out because I would like to be able to accept your paper. I am sorry to ask for further work from you but I would not expect to return this manuscript to the reviewers.

Please ensure that you clearly highlight all changes made in the revised manuscript. Please avoid using 'Tracked changes' in Word files as these are lost in PDF conversion.

I should be grateful if you would also provide a point-by-point response detailing how you have dealt with the points raised by the reviewers in the 'Response to Reviewers' box. Please attend to all of the reviewers' comments. If you do not agree with any of their criticisms or suggestions please explain clearly why this is so.

Reviewer 1

Advance summary and potential significance to field

The authors made significant changes, with additional data from SEM imaging of bristles and quantification of the tip localization of SpnF in the pupal bristles. I apologize to the authors for mentioning IKK epsilon, which should be SpnF.

I have reread the whole manuscript. The purpose of this work is to report the functions of BicDR related to the dynein adaptor molecule BicD. To this end, the authors examined genetic interaction of BicD and BicDR in mechanosensory hair development. They provided evidence, based on the analyses of adult bristles, that the two genes interact. They also showed the pupal bristles of trans-heterozygotes of BicDR and BicD alleles showed disorganization of actin bundles. They also showed the IP-mass-spec analysis of proteins associated with BicD in the embryo. One candidate molecule was EF1gamma, involved in protein translation. Those results support the hypothesis that the two genes act together in bristle development, through the control of mRNA transport. This is consistent with the previously reported report of BicD function in dynein-dependent mRNA transport.

Although the data set is consistent with the hypothesis, it does not exclude other possible explanations. My concern is that the molecular characterization of BicDR, in its activity to interact with dynein, BicD, or EF1gamma, is not strong enough.

Comments for the author

Line 121. It was stated 12 out of 18 females and 2 out of 8 males showed abnormal bristle phenotype. In Fig.
1F. n=30 is indicated. Is this the number of total adult flies counted? It does not much the number in the text.

Line 171 “Bristles contain actin filament bundles that are clearly seen, particularly in the proximal region close to the base (Fig. 2D).” Confusing text. Figure 2D showed an SEM image of the cuticle surface, not actin staining. The following description should be rewritten. Perhaps one way to clarify this point is to present actin localization in the bristle first and describe the adult cuticle ridge patterns, which are known to be influenced by actin bundle distribution in the pupal stage.

Figure 3. The quality of BicDR:GFP staining in Fig. 3C is of marginal quality, and it would be difficult to discuss the relationship of BicDR:GFP positive and Ase positive cells.

Line 223. Fig. 4C should be Fig. 3C?

© 2023. Published by The Company of Biologists under the terms of the Creative Commons Attribution License (https://creativecommons.org/licenses/by/4.0/).

Reviewer 2

Advance summary and potential significance to field

In this manuscript, the authors have shown a role for BicDR in the assembly of the macrochaetae (large bristles) of the adult fly. They have shown that in the absence of the related protein BicD, BicDR loss or reduction enhances the bristle defects.The authors have provided evidence that localization of Spn-F and Rab6 are disrupted in the double mutant combinations and they have discovered proteins that physically interact with BicDR. They follow up on one of these proteins, EF1gamma showing that it genetically interacts with BicD and BicDR to enhance bristle defects. The authors suggest that whereas BicD is involved in trafficking over long distances, BicDR may be involved in short range trafficking.

Comments for the author

This is a much tighter and more convincing study as a result of additional experiments and other modifications. As currently presented, it is much easier to follow the phenotypic changes associated with loss of BicDR in the different mutant backgrounds. The inclusion of the SEM analysis was a very positive addition to the study.

Minor points.

Figure 2A,C - in the color code for the histogram, the authors should not include a variety of symbols - just use a rectangle showing what genotype each of the colors represents. Also remove the "Lorem ipsum" from the figure.

Lines 223, 224 - I would leave out the part about the trachea - I don't think phenotypes were explored for any genotype beyond the BicDR delta71 homozygotes and the observed defects could be due to anything on the chromosome that was also homozygosed in this condition. Moreover, there is no evidence of trachea expression (the segmental staining observed with the GFP-tagged protein is not trachea). The authors did say (in the response) that they did not observe defects in the salivary gland, but I did not see this in the manuscript - perhaps it could be mentioned at this point in the paper.

Discussion - the authors did not test this gene for a role in life outside the laboratory setting - so this comment at the beginning of the discussion should be removed. I think that most of us in the field appreciate that genes with no obvious phenotypes in the lab setting when removed probably have some function or provide some advantage in the wild.

Second revision

Author response to reviewers' comments

Reviewer 1 Comments for the Author:
Line 121. It was stated 12 out of 18 females and 2 out of 8 males showed abnormal bristle phenotype. In Fig. 1F. n=30 is indicated. Is this the number of total adult flies counted? It does not much the number in the text.

➔ Thank you for picking this up. We made the change in Fig. 1F to n=26. We then also realized that the Figure legend to Fig. 1E-F was still an old version. This has now been corrected, too. (The sentence with the correct n=26 was in (E) and under (F) we had an old sentence from the previous version).

Line 171 “Bristles contain actin filament bundles that are clearly seen, particularly in the proximal region close to the base (Fig. 2D).” Confusing text. Figure 2D showed an SEM image of the cuticle

© 2023. Published by The Company of Biologists under the terms of the Creative Commons Attribution License (https://creativecommons.org/licenses/by/4.0/).

surface, not actin staining. The following description should be rewritten. Perhaps one way to clarify this point is to present actin localization in the bristle first and describe the adult cuticle ridge patterns, which are known to be influenced by actin bundle distribution in the pupal stage.

➔ It is true, that the SEM pictures visualize the surface (the gold coating in this case) and not directly the actin bundles. The surface snuggles around the actin bundles, and this allows us to use the SEM to judge the underlying structure. Although we wish to clarify that we did not write “actin staining”, we got the point and changed the phrasing by referring to these surface structures as “ridges” and “ridges with (the) bundle structure”. The new phrasing seems now precise and still understandable. As also mentioned by this reviewer, the connection with the underlying actin bundle structure is common knowledge. This is one reason why we feel that it will not improve the manuscript if we move the actin staining of the pupal bristle into Figure 2. The second one is that the actin staining of the pupal bristle would not make a better case that the SEM picture of the adult bristle ridges serve as a proxy for the actin filament bundles. With the present phrasing, we feel that it is more coherent to leave the actin staining in Figure 4 with the other stainings of pupal bristles.

Figure 3. The quality of BicDR:GFP staining in Fig. 3C is of marginal quality, and it would be difficult to discuss the relationship of BicDR:GFP positive and Ase positive cells.

➔ We had pointed out this difficulty in the manuscript, too. Figure 3 shows the expression of the endogenously tagged BicDR::GFP. Its expression is generally low, making this analysis difficult. In the end, we did not focus on this expression pattern because we had identified the macrochaetae phenotype that pointed us to the cells where we could study BicDR’s function. The initial characterization of the expression could still become useful for other groups working on these tissues or BicDR. They would then have to study in more detail the metameric expression pattern shown in Fig. 3C. For us, it turned out to be a good choice to move to the related pupal tissue.

Line 223. Fig. 4C should be Fig. 3C?
➔ We corrected Fig. 4C (now on line 221) to Fig. 3C - thank you!

Reviewer 2 Advance Summary and Potential Significance to Field:
In this manuscript, the authors have shown a role for BicDR in the assembly of the macrochaetae (large bristles) of the adult fly. They have shown that in the absence of the related protein BicD, BicDR loss or reduction enhances the bristle defects.The authors have provided evidence that localization of Spn-F and Rab6 are disrupted in the double mutant combinations and they have discovered proteins that physically interact with BicDR. They follow up on one of these proteins, EF1gamma, showing that it genetically interacts with BicD and BicDR to enhance bristle defects. The authors suggest that whereas BicD is involved in trafficking over long distances, BicDR may be involved in short range trafficking.

Reviewer 2 Comments for the Author:

Minor points.

Figure 2A,C - in the color code for the histogram, the authors should not include a variety of symbols - just use a rectangle showing what genotype each of the colors represents. Also remove the "Lorem ipsum" from the figure.

➔ We have made these changes.

Lines 223, 224 - I would leave out the part about the trachea - I don't think phenotypes were explored for any genotype beyond the BicDR delta71 homozygotes, and the observed defects could be due to anything on the chromosome that was also homozygosed in this condition. Moreover, there is no evidence of trachea expression (the segmental staining observed with the GFP-tagged protein is not trachea). The authors did say (in the response) that they did not observe defects in the salivary gland, but I did not see this in the manuscript - perhaps it could be mentioned at this point in the paper.

➔ We have made changes precisely as suggested. It now reads like this.

© 2023. Published by The Company of Biologists under the terms of the Creative Commons Attribution License (https://creativecommons.org/licenses/by/4.0/).

"Because initial experiments did not reveal a function of *BicDR* in salivary glands but identified defects in the adult bristles of the mutants, we focused on the function of *BicDR* in bristle development."

Discussion - the authors did not test this gene for a role in life outside the laboratory setting - so this comment at the beginning of the discussion should be removed. I think that most of us in the field appreciate that genes with no obvious phenotypes in the lab setting when removed probably have some function or provide some advantage in the wild.

➔ We have removed this.

Third decision letter

MS ID#: JOCES/2023/261408

MS TITLE: Role of BicDR in bristle shaft construction and support of BicD functions

AUTHORS: Aleksandra Jejina, Yeniffer Ayala, Dirk Beuchle, Thomas C. Hoehener, Ruth E. Doerig, Paula Vazquez-Pianzola, Greco Hernandez, and Beat Suter
ARTICLE TYPE: Research Article

I am happy to tell you that your manuscript has been accepted for publication in Journal of Cell Science, pending standard ethics checks. I have made this decision without referring again to the reviewers so there are no new comments on the manuscript from them. I accept your arguments in the rebuttal and congratulate you on some nice work.

© 2023. Published by The Company of Biologists under the terms of the Creative Commons Attribution License (https://creativecommons.org/licenses/by/4.0/).
